# Rapid Phenotypic Antibiotics Susceptibility Analysis by a 3D Printed Prototype

**DOI:** 10.1002/advs.202308806

**Published:** 2024-03-26

**Authors:** Oliver Riester, Lars Kaiser, Stefan Laufer, Hans‐Peter Deigner

**Affiliations:** ^1^ Institute of Precision Medicine Furtwangen University Jakob‐Kienzle‐Strasse 17 78054 Villingen‐Schwenningen Germany; ^2^ Institute of Pharmaceutical Sciences Department of Pharmacy and Biochemistry Eberhard‐Karls‐University Tuebingen Auf der Morgenstelle 8 72076 Tuebingen Germany; ^3^ Tuebingen Center for Academic Drug Discovery & Development (TüCAD2) 72076 Tuebingen Germany; ^4^ IFIT Cluster of Excellence EXC 2180 ‘Image‐Guided and Functionally Instructed Tumor Therapies’ University of Tuebingen 72076 Tuebingen Germany; ^5^ Faculty of Science Eberhard‐Karls‐University Tuebingen Auf der Morgenstelle 8 72076 Tuebingen Germany; ^6^ EXIM Department Fraunhofer Institute IZI (Leipzig) Schillingallee 68 18057 Rostock Germany

**Keywords:** 3D printer, antibiotic resistances, antibiotic susceptibility testing, no preculture, phenotypic, screen printed electrodes

## Abstract

One of the most important public health concerns is the increase in antibiotic‐resistant pathogens and corresponding treatment of associated infections. Addressing this challenge requires more efficient use of antibiotics, achievable by the use of evidence‐based, effective antibiotics identified by antibiotic susceptibility testing (AST). However, the current standard method of phenotypic AST used for this purpose requires 48 h or more from sample collection to result. Until results are available, broad‐spectrum antibiotics are used to avoid delaying treatment. The turnaround time must therefore be shortened in order for the results to be available before the second administration of antibiotics. The phenotypic electrochemical AST method presented here identifies effective antibiotics within 5–10 h after sampling. Spiked serum samples, including polymicrobial samples, with clinically relevant pathogens and respective concentrations commonly found in bloodstream infections (*Escherichia coli*, *Staphylococcus aureus*, *Klebsiella pneumoniae*, *and Pseudomonas aeruginosa*) are used. Direct loading of the test with diluted serum eliminates the need for a pre‐culture, as required by existing methods. Furthermore, by combining several electrochemical measurement procedures with computational analysis, allowing the method to be used both online and offline, the AST achieves a sensitivity of 94.44% and a specificity of 95.83% considering each replicate individually.

## Graphical abstract

Rapid and targeted antibiotic treatment of multi‐resistant pathogens conserves reserve antibiotics and improves the success of treatment. A rapid 3D‐printed prototype for the electrochemical detection of antibiotic susceptibility directly from serum, including polymicrobial samples, is presented. Compared to the current standard, a considerable time reduction has been achieved, enabling targeted antibiotic treatment on the day of sampling.

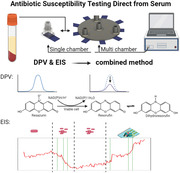

## 1 Introduction

Treating infections caused by antibiotic‐resistant (AR) pathogens displays an increasing challenge and is, according to the WHO, a growing health problem worldwide that could lead to 10 million deaths per year by 2050, if no action is taken.^[^ ^1^ ^]^ As of 2019, infections with AR pathogens were responsible for a worrying number of deaths worldwide, accounting for ≈4.95 million (64 per 100 000) deaths associated with AR pathogens and 1.27 million (16.4 per 100 000) deaths directly attributable to infections with AR pathogens.^[^ ^2^ ^]^ In particular, the fact that pathogens resistant to new antibiotics are often detected in clinics within a year after their approval,^[^ ^3^ ^]^ as well as the correlation between excessive use and the development of new resistances,^[^ ^4^ ^]^ highlights the necessity of responsible and restrictive use of antibiotics. Although the causal relationship between excessive antibiotic use and the emergence of resistance has been acknowledged for some time, antibiotic use is increasing rather than decreasing,^[^ ^5^, ^6^ ^]^ particularly in the veterinary sector, where estimated antimicrobial consumption is projected to increase by 8% between 2020 and 2030, based on current trends.^[^ ^7^ ^]^ In addition to reducing the likelihood of new resistances emerging, the targeted use of antibiotics with confirmed efficacy has the advantage of improving treatment outcomes.^[^ ^8^, ^9^ ^]^ However, in order to use the most appropriate and effective antibiotic, one must first know which is effective against the pathogen present, thus requiring antimicrobial susceptibility testing (AST). The current state of the art involves blood culture as the first step, followed by isolation of pathogens and finally AST.^[^ ^10^ ^]^ As a result, the turnaround time from sample collection to AST result is typically 48 h or more, with blood culture and isolation of potential pathogens taking 24 to 48 h and the actual AST taking 16 to 24 h for gold standard methods or 4 to 8 h for automated methods.^[^ ^10^, ^11^ ^]^ However, it would be desirable to obtain an AST result within 8 h or less after sample collection in order to be able to administer a proven effective antibiotic to the patient after the initial antibiotic administration. For this reason, a wide variety of technologies and processes are being tested and further developed to determine their suitability for meeting the desired turnaround time while maintaining the same quality as the current gold standard.

AST are classified in two categories: phenotypic and genotypic. Phenotypic approaches, the current gold standard, study the effect of an administered antibiotic on a bacterial population by measuring its metabolic activity or bacterial growth and thus detect the direct effect of the antibiotic. Genotypic approaches, on the other hand, do not detect the direct effect of the antibiotic, rather they test for the presence of genes that are required for resistance. This approach has the advantage that, in theory, there is no need for prior cultivation, although this still represents a challenge in practice.^[^ ^12^ ^]^ Shifman et al.^[^ ^13^ ^]^ used a reverse‐transcriptase polymerase chain reaction (RT‐PCR) to quantify RNA Markers of Yersinia pestis accountable for ARs. They were able to determine the minimum inhibitory concentration of doxycycline within 7 h after positive blood culture, a significant advantage over the CLSI standard of 24 h, especially for *Yersinia pestis*, whose slow ex vivo growth was specifically pointed out by the authors. A disadvantage of PCR‐based methods is that the primers targeting the resistance gene must be chosen specifically in advance. This problem can be addressed by whole genome sequencing (WGS) approaches, which are becoming increasingly attractive as next‐generation sequencing advances. For example, using nanopore metagenomics, Charalampous et al.^[^ ^14^ ^]^ demonstrated that it is possible to detect the pathogen and the resistance genes of a bacterial lower respiratory tract infection within 6 h. Nevertheless, the European Committee on Antimicrobial Susceptibility Testing (EUCAST) stated in 2017 that the available evidence for WGS as an AST tool is either insufficient or nonexistent and therefore inappropriate for clinical decision making.^[^ ^12^ ^]^ They also pointed out the urgent need for a single database of all known resistance genes/mutations to facilitate comparison between different systems and bioinformatic tools. These statements are also supported by a study from Rebelo et al.^[^ ^15^ ^]^ in which they compared 500 bacterial isolates from Danish clinical microbiology laboratories tested with WGS (Illumina NextSeq, genotypic) and standard broth microdilution (phenotypic). Concordance was observed in 91.7 % of all cases, with the remaining 8.3 % divided as follows: 6.2 % were phenotypically susceptible isolate‐antimicrobial combinations that possessed resistance genes but did not exhibit effective resistance, and 2.1 % had phenotypic resistances that were not detected by WGS, representing 26.4 % of all phenotypic resistances present that were not detected by WGS. This highlights that, given the current state of the art, genotypic tests, particularly WGS‐based tests, often require phenotypic verification to provide clinically relevant conclusions.^[^ ^16^ ^]^ Therefore, phenotypic AST tests will remain irreplaceable as the standard for the foreseeable future, making it vital to reduce their turnaround time.

To shorten the turnaround time of phenotypic AST, it is particularly helpful to avoid the upstream blood culture, thus requiring tests to be loaded directly with the patient sample or a dilution thereof and the bacterial concentrations present therein. The majority of AR‐associated deaths are caused by lower respiratory or bloodstream infections ^[^ ^2^ ^]^ and sputum or serum specimens contain approximately >10^4 [^ ^17^, ^18^ ^]^ or < 10^3^ CFU/ml,^[^ ^19^, ^20^ ^]^ respectively. Thus, an appropriate detection method should be able to provide results ideally within the first 8 h of testing under these starting conditions. A variety of detection methods have been described in the literature for rapid phenotypic AST, such as detection by fluorescence,^[^ ^21^, ^22^ ^]^ luminescent measurement of extracellular ATP,^[^ ^23^ ^]^ microscopic detection of cell growth,^[^ ^24^ ^]^ antibody‐modified magnetic nanoparticles,^[^ ^25^ ^]^ electrochemical measurements by differential pulsed voltammetry (DPV) ^[^ ^26^, ^27^, ^28^ ^]^ or electrochemical impedance spectroscopy (EIS).^[^ ^29^ ^]^

However, most of them were demonstrated only in buffer, growth medium, or artificial urine, and often at high initial concentrations of 10^5^ CFU/ml or more, requiring prior incubation. Some methods also destroy the sample during the measurement, e.g. by lysing the bacteria, which leads to an increased number of samples if a time course and no endpoint determination is to be recorded.

**Figure 1:**
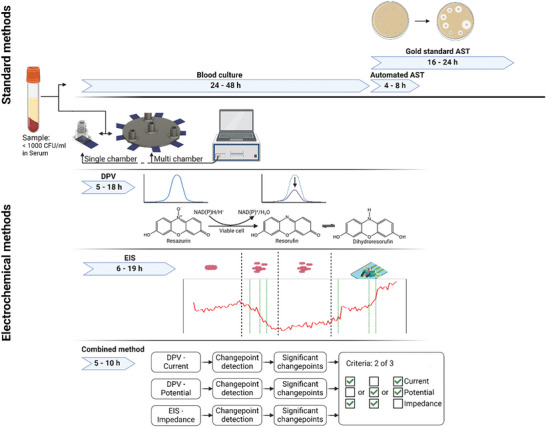
Overview of AST with respective time intervals using standard methods or the here demonstrated electrochemical methods. The rapid AST platform in a 3D printed microfluidic device can be used as a single chamber or a multi‐chamber version. Measurement of antimicrobial susceptibility is based on electrochemical impedance spectroscopy (EIS) and the metabolic reduction of resazurin to resorufin analyzed by differential pulse voltammetry (DPV).

Herein, we demonstrate a 3D‐printed phenotypic rapid AST system based on a combination of DPV and EIS measurements. To our knowledge, this study is the first demonstration of a rapid AST system that provides results from spiked serum samples at 1000 CFU/ml in <10 h after sample collection (**Figure** **1**). The approach combines EIS measurements with DPV measurements of Resazurin, which is metabolized by living bacteria to resorufin.^[^ ^30^ ^]^ The assay was tested for seven clinically relevant bacterial strains with different resistance profiles to kanamycin and oxytetracycline, which were selected as example antibiotics due to their widespread resistance ^[^ ^31^, ^32^ ^]^ and their correspondence with the resistance/susceptibility profile of the bacterial strains used. Included were most common pathogens of bloodstream infections, such as *Escherichia coli* (*E. coli*), *Staphylococcus aureus* (*S. aureus*), *Klebsiella pneumonia* (*K. pneumonia*), and *Pseudomonas aeruginosa* (*P. aeruginosa*).^[^ ^33^ ^]^ Furthermore, it was demonstrated that the 3D‐printed system can be scaled up to multiple chambers and can also be complemented with a suitable microfluidic channel system to ensure user‐friendly loading.

## 2 Results and Discussion

### 2.1 Combining 3D Printed Microfluidic Device Designs with Embedded SPEs

**Figure 2:**
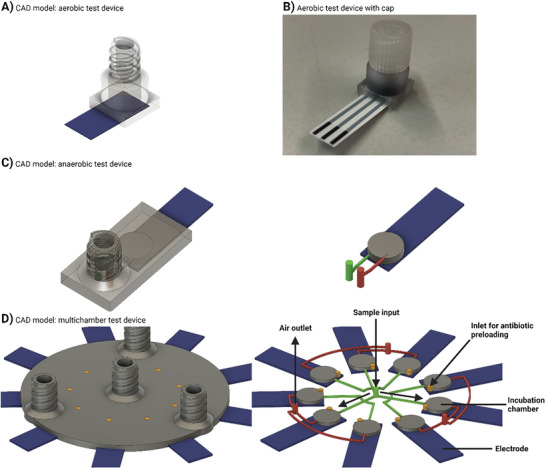
3D printed device designs. 3D‐printed devices with integrated SPEs were fabricated from PMMA in various designs. A) CAD model of aerobic design with headspace for oxygen exchange. B) Image of aerobic test device with implemented IS‐C electrode and sealed with Luer lock cap. CAD model of C) single‐chamber and D) multi‐chamber versions of the anaerobic design with respective microfluidic distribution structures. Shown are microfluidic structures for filling with sample (green), venting air present (red), and preloading antibiotics (orange). Arrows indicate flow direction during sample loading. For images of the 3D‐printed anaerobic and multi‐chamber designs, the reader is referred the supplementary materials.

The devices were designed such that commercial screen‐printed electrodes (SPEs) could be embedded within the test system. By combining the SPEs with a 3D‐printed microfluidic device, a closed system was developed, minimizing evaporation and allowing test intervals of more than 16 h. Several designs were created that demonstrate the flexibility of the concept to meet application requirements. For example, devices were developed for anaerobic and aerobic culture conditions in single and multi‐chamber versions (**Figure** **2**). The anaerobic and multi‐chamber devices were only tested for tightness and ease of filling in order to provide a proof of concept (Figure S1, Supporting Information). AST experiments were performed in single‐chamber aerobic devices (Figure 2A,B), with multiple devices being measured in parallel. The designs can be further adapted to suit the desired test conditions and the number of antibiotics or concentrations to be tested. For example, the multi‐chamber version can be adapted to an aerobic design by removing the vent channels (Figure 2D, shown in red) and replacing the top layer of the chambers with a gas permeable membrane.

### 2.2 Antibiotic Susceptibility Test by Agar Diffusion Assay – Gold Standard

**Figure 3:**
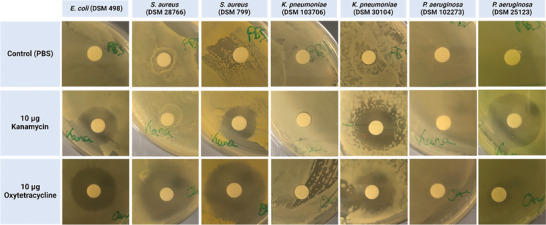
Antibiotic susceptibility test by agar diffusion test. Indicated bacterial strains are tested for susceptibility to the antibiotics kanamycin and oxytetracycline at concentrations of 10 µg per disc. Susceptibility to antibiotics is indicated by the formation of an inhibition zone surrounding the discs. PBS was included as control.

To enable a comparison of the newly developed method with the current gold standard, the used strain‐antibiotic combinations were evaluated using the agar diffusion test, displayed in **Figure** **3**. The test was performed as reference AST with 21 strain‐antibiotic combinations, including 7 untreated, 7 treated with kanamycin, and 7 treated with oxytetracycline. The reference test demonstrated growth in all 7 untreated samples, resistance in 6 combinations, and susceptibility in 8 combinations. The observed resistances were mostly consistent with data available in the Bacdive library.^[^ ^34^ ^]^ The only differences were observed for the *P. aeruginosa* strains, as *P. aeruginosa* (DSM 102273) should be sensitive to tetracycline (no entry for oxytetracycline) according to the library, which was not observed for oxytetracycline, chemically similar to tetracycline. In addition, *P. aeruginosa* (DSM 25123) is not listed as resistant in the library, but showed resistance or high tolerance to oxytetracycline. These differences may also be due to differences in the concentrations tested, as they were chosen to be comparable to the ones used in the electrochemical experiments.

### 2.3 Antibiotic Susceptibility Test by Electrochemical Detection

**Figure 4:**
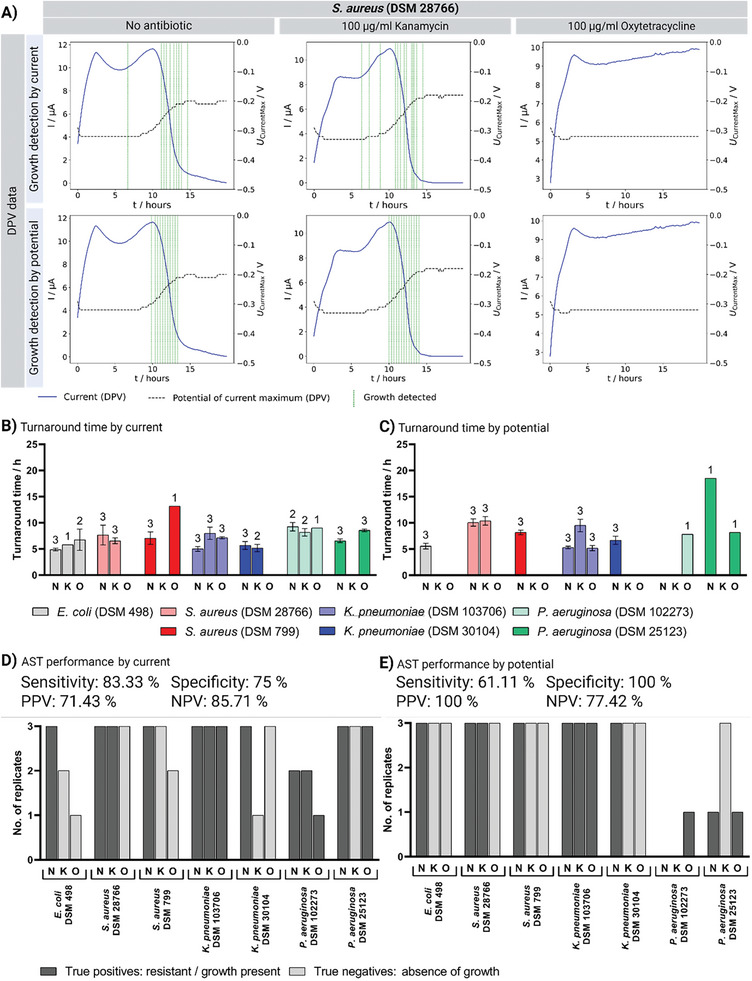
Electrochemical measurement and bioinformatic analysis of antibiotic susceptibility by DPV. Bacterial growth of pathogens (1000 CFU/ml in serum corresponding to 50 CFU/measuring chamber) with 100 µg ml^−1^ kanamycin (K), 100 µg ml^−1^ oxytetracycline (O), or without antibiotic (N) detected by differential pulsed voltammetry (DPV) via current intensity or potential shift. A) Representative replicates for DPV measurement of MRSA (DSM 28766) with growth detected by current or potential. Turnaround time (mean ± SD) until growth was detected by B) current or C) potential. Number above bars indicates replicates that detected growth (*n* = 3, independent devices). Performance of AST by D) current or E) potential compared to agar diffusion assay (Figure 3) with true positives (resistant to treatment/exhibits growth) and true negatives (susceptible to treatment/exhibits no growth).

**Figure 5:**
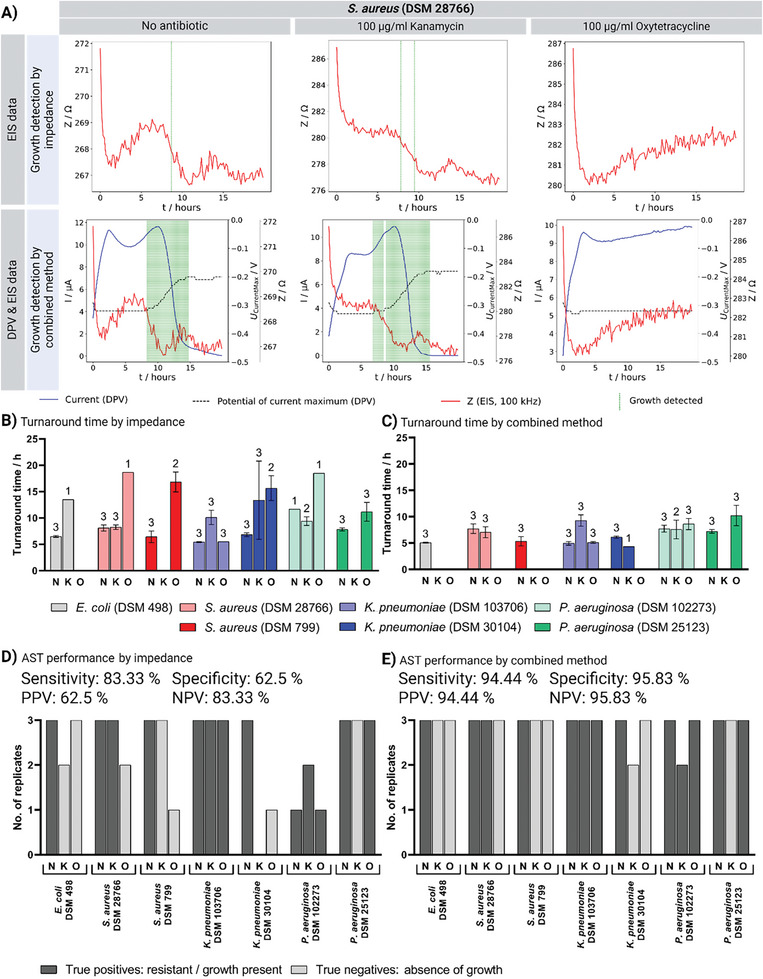
Electrochemical measurement and bioinformatic analysis of antibiotic susceptibility by EIS or combined method. Bacterial growth of pathogens (1000 CFU/ml in serum corresponding to 50 CFU/measuring chamber) with 100 µg ml^−1^ kanamycin (K), 100 µg ml^−1^ oxytetracycline (O), or without antibiotic (N) detected by electrochemical impedance spectroscopy (EIS) or a combined method of EIS and DPV. A) Representative replicates for EIS and DPV measurement of MRSA (DSM 28766) with growth detected by impedance or combined method. Turnaround time (mean ± SD) until growth was detected by B) impedance or C) combined method. Number above bars indicates replicates that detected growth (*n* = 3, independent devices). Performance of AST by D) impedance or E) combined method compared to agar diffusion assay (Figure 3) with true positives (resistant to treatment/exhibits growth) and true negatives (susceptible to treatment/exhibits no growth).

Phenotypic AST using electrochemical methods for the detection of bacterial growth have several advantages, especially when compared to fluorescence measurements, such as comparable sensitivity and selectivity, low cost, low power requirements, multiple measurement methods, and compatibility with microfabrication technology.^[^ ^35^ ^]^ In particular the possibility to combine several measurement methods in one device and perform them simultaneously for one sample offers immense potential. In the following, the detection of bacterial growth (or the absence when susceptible to antibiotic) for clinically relevant bacterial strains in spiked plasma samples using DPV and EIS in combination with computational analysis is demonstrated. Representative plots for multi‐resistant *Staphylococcus aureus* (MRSA, DSM 28766) are shown as examples in **Figures** **4** and **5**. MRSA was selected as an example strain as it shows resistance to kanamycin and sensitivity to oxytetracycline. For representative plots of further tested bacterial strains (*E. coli* (DSM 498); *S. aureus* (DSM 799); *K. pneumoniae* (DSM 103706); *K. pneumoniae* (DSM 30104); *P. aeruginosa* (DSM 102273); *P. aeruginosa* (DSM 25123)) the reader is referred to Figures S2–S8 (Supporting Information). The ASTs performance (sensitivity, specificity, positive predictive value (PPV), and negative predictive value (NPV)) was evaluated using the agar diffusion assay (Figure 3) as reference. Furthermore, the turnaround time for detection of growth was analyzed using each method individually or in combination (Table S1, Supporting Information; **Figures** **4** and **5**). The electrochemical antibiotic susceptibility test was performed 68 times, including five control samples without bacteria and the same strain‐antibiotic combinations as for the agar diffusion assay, each with three independent replicates.

#### 2.3.1 Differential Pulsed Voltammetry (DPV)

The use of DPV for the measurement of resazurin metabolization is already described in the literature.^[^ ^27^, ^28^ ^]^ However, to our knowledge, the method has never been demonstrated in plasma or serum samples with realistic bacterial concentrations suitable for bloodstream infection. Diluted samples of spiked serum (1000 CFU/ml) were tested with an ItalSens‐Carbon (IS‐C) SPE, resulting in 250 CFU/ml in 25/75% FCS/LB. It was observed that the current was unstable in the first 3 to 4 h and the absolute initial values varied from device to device at the same initial resazurin concentration (Figure 4A). The observed increase in current (*I*) during the first 3 to 4 h was likely the result of protein adsorption and equilibration of the reference electrode caused by wetting effects and was observed for all bacterial strains and controls. However, the effect did not influence the measurement, as within the first few hours the growth of the bacteria is still too low to be detected. In addition, it was shown (Figure S12, Supporting Information), that this effect can be reduced by pre‐wetting the electrodes in advance. Although the initial current increase did not interfere with the measurement itself, pre‐equilibrium values were excluded and the analysis was performed with the first derivative, as the increase was always detected as a changepoint by the detection algorithm.

The analysis of the first derivative has two advantages in this respect: first, the offset of the individual instruments becomes less influential and second, the signal change, which serves as evidence of bacterial growth, becomes more comparable for different samples. The first derivative of *I* can assume both positive and negative values as a result of bacterial growth. Negative values (e.g., Figure 4A: *S. aureus* – No antibiotic between 10 and 14 h) can be interpreted as a decrease in resazurin concentration due to its metabolization, as shown in Figures S9 and S10 (Supporting Information) for different resazurin concentrations. Positive values (e.g., Figure 4A: *S. aureus* – No antibiotic between 6 and 9 h), on the other hand, can be explained by a decrease in pH as described by Cakir et al.,^[^ ^30^ ^]^ e.g., as a result of accumulated metabolites during bacterial growth. The presence of additional protons increases the measured current of the 2‐electron transfer reaction from resazurin to resorufin and the potential of the peak maximum in the course of the experiment, leading to an initial increase in the current until the effect is superimposed by the decrease in the resazurin concentration. The decrease in current in samples with bacterial growth is therefore not solely due to the decrease in resazurin concentration, but also partly due to the aforementioned peak shift, as the evaluation was carried out constantly at −0.3 V, even if the potential of the peak maximum had shifted. In addition to the current intensity, however, the peak shift can also be used as a parameter for determining bacterial growth.

Bioinformatic analysis via current intensity using change point detection resulted in growth detection after 4.89 ± 0.21 h up to 13.17 h, depending on the strain‐antibiotic combination (Figure 4B). In some cases, growth was also delayed by the addition of antibiotics, e.g. when *K. pneumoniae* (DSM 107306) was tested for susceptibility to kanamycin (Figure 4B,C; Figure S5, Supporting Information), indicating a partial effect of the antibiotic but not sufficient to completely stop growth. In the 42 tests performed excluding controls, analysis via current intensity detected 21 (15 true positives, 6 false positives) resistant and 21 (18 true negatives, 3 false negatives) sensitive strain‐antibiotic combinations, resulting in a sensitivity of 83.33% and a specificity of 75% (Figure 4D). When using the peak maximum potential as a parameter for change point detection, growth was detected between 5.17 ± 0.41 h and 18.5 h (Figure 4C), and thus slower than detection via current. Analysis via potential resulted in the detection of 11 (11 true positives, 0 false positives) resistances and 31 (24 true negatives, 7 false negatives) susceptibilities, resulting in a lower sensitivity of 61.11% but a higher specificity of 100% (Figure 4E).

#### 2.3.2 Electrochemical Impedance Spectroscopy (EIS)

In order to improve the performance of the electrochemical AST and to detect slowly metabolizing or biofilm‐forming pathogens, the DPV method was supplemented by EIS, a method that can be performed with the same experimental setup. The principle of the EIS measurement method for bacterial growth can produce both increasing and decreasing curve patterns for measured impedance over time, which are explained in the literature as follows: an increase in impedance is due to a decrease in accessible electrode surface area as a result of a bacterial layer on the surface (e.g., biofilm formation), whose cell membranes act as an insulating layer, decreasing the accessible area for electron transfer.^[^ ^36^ ^]^

The effect of impedance reduction is described, for example, by Asami et al.,^[^ ^37^ ^]^ stating that living bacteria with intact cell membranes behave like electrical capacitors in solution. By increasing in numbers, the bacteria increase the capacitive characteristics of the dielectric and thereby influence the measured capacitance (*C*) and impedance (*Z*). According to equation 1, where *i* is the imaginary unit and ω the angular frequency, an increase in *C* results in a decrease of *Z*.

(1) $$Z\;=\frac{1}{i\omega C}\;$$

The frequency of the alternating current applied during the EIS measurement also determines which components or properties, such as cell size, cell membrane, or periplasmic space, have an influence on the measurement signal.^[^ ^38^, ^39^ ^]^ In theory, the effects described result in various plausible signal changes. However, for all seven strains tested it became apparent that they caused a reduction in impedance during growth, which indicates planktonic growth according to the second effect. For some antibiotic‐strain combinations (e.g., oxytetracycline – *K. pneumoniae*, Figure S5, Supporting Information), a subsequent increase in impedance is observed after a longer test period, possibly indicating the formation of a biofilm on the electrode according to the first effect. Using impedance (*Z*) at 100 kHz as a parameter for bioinformatic analysis to detect bacterial growth resulted in turnaround times ranging from 5.44 ± 0.08 h up to 18.67 h, depending on the pathogen. Thereby, 24 (15 true positives, 9 false positives) resistant and 18 (15 true negatives, 3 false negatives) sensitive strain‐antibiotic combinations were detected, which results in a sensitivity of 83.33% and a specificity of 62.5%.

#### 2.3.3 Analysis by Combined Method

As previously demonstrated, growth detection based solely on current, potential, or impedance parameters have their advantages and disadvantages. For example, detection via current showed the fastest turnaround times, but only mediocre sensitivity (83.33%) and specificity (75%), as well as low signal changes in pathogens with slow metabolism such as *P. aeruginosa* (Figures S7 and S8, Supporting Information). For pathogens with slow metabolism, detection by impedance was advantageous as the measurement principle is not based on metabolic activity. However, the specificity was significantly lower with 62.5%, while it was 100% for detection via potential. In order to use the advantages of all parameters and measurement principles and thus compensate for their disadvantages, a combined analysis was performed using all parameters. The combination of different electrochemical measurement methods benefits the AST assay by incorporating all favorable signal changes and cross‐checking the results obtained by the different parameters. This results in a reduction of false positives and false negatives, thus increasing the sensitivity and selectivity of the test. Using the combined method (significant changepoint for 2 of 3 parameters over the last hour), 94.44% sensitivity and 95.83% selectivity were achieved at turnaround times of 4.94 ± 0.28 to 10.22 ± 1.59 h, based on a reduction in the number of false negatives and false positives to 1 out of 42 tests, respectively. The method is therefore suitable for identifying an effective antibiotic within 8–24 h after sampling and significantly reduces the 28–72 h typically required by standard methods.

## 3 Conclusion

In summary, an approach for efficient and reliable antibiotic susceptibility testing is presented, which is already close to a prototype with clinical utility. It demonstrates the combination of different electrochemical measurement methods in a device that benefits from 3D‐printed microfluidic devices and leads to reproducible and rapid results, even for clinically problematic pathogens. The AST enables treatment of patients with evidence‐based, effective antibiotics, improving treatment outcomes while conserving available reserve antibiotics by reducing future antibiotic resistance. The assay presented here was used to detect resistance to kanamycin and oxytetracycline in seven strains, including *E. coli* and two strains each of *S. aureus*, *K. pneumoniae*, and *P. aeruginosa* with different resistance profiles. Bacterial growth (or lack thereof) was detected after 4.94 ± 0.28 to 10.22 ± 1.59 h under realistic conditions of a bloodstream infection, resulting in the resistance profile for each sample. For each individual replicate, a sensitivity of 94.44% and a specificity of 95.83% was obtained, or 100% for both when the majority outcome of each triplicate was considered. The time saving is achieved in particular by cultivating the bacteria (blood culture requires 24–48 h by default) and the actual AST (4–24 h by default) in a single step. Compared to other AST systems (**Table** **1**), the turnaround time was significantly reduced, allowing an effective antibiotic to be identified at the time of the second antibiotic administration, thereby significantly improving the treatment outcome. Furthermore, the use of broad‐spectrum antibiotics and the total amount of administered antibiotics can be reduced, thus limiting the emergence of further resistances. It was also pointed out that the assay has the potential to be used in multi‐chamber systems with an appropriate microfluidic distribution structure for easy filling. This would not only allow the serum sample to be tested for different antibiotics simultaneously and in principle for polymicrobial infections (Figures S13 and S14, Supporting Information), but also allow the minimum inhibitory concentration of the antibiotic in question to be determined by testing different concentrations of antibiotics (Figure S15, Supporting Information). The authors also note that the electrodes used are unmodified, commercially available electrodes that could be exchanged for optimized electrodes with higher sensitivity to further improve the turnaround time and robustness of the test system. The properties of the electrodes, such as the material of the working and reference electrodes or modifications of the surface, as well as the properties of the medium have an influence on the respective measurement results, thereby a direct transfer of the results and parameters to be analyzed to systems with other electrodes should be validated individually in each case.

**Table 1:** Comparison of the demonstrated phenotypic AST system with commercial (Vitek2 and BD Phoenix) and experimental systems.

AST system	This study	Vitek2 ^[^ ^40^ ^]^	BD Phoenix ^[^ ^40^ ^]^	Hannah et al. ^[^ ^41^ ^]^	Baltekin et al. ^[^ ^24^ ^]^	Azizi et al. ^[^ ^22^ ^]^
Measurement principle	Electrochemical (DPV, EIS)	Turbidity	Colorimetric redox indicator	Electrochemical (EIS)	Microscopy	Fluorescence
Sample origin	Serum	Respiratory, blood, eye	Respiratory, blood, eye	Growth medium	Urine or growth medium	Growth medium
Preculture needed?	Direct	Preculture 18–24 h	Preculture 18–24 h	Preculture overnight (12–18 h)	Preculture 2 h	Preculture overnight (12 h)
Sample preparation	Dilute 1/4 with growth medium	“McFarland standard of 0.5 to 0.63 in 0.45% sodium chloride”	“25 µl was transferred to AST broth to obtain a final inoculum density of ≈5 × 10^5^ CFU/ml”	“Bacteria from the overnight cultures were directly pipetted onto the gels on the electrode surface”	During chip loading, bacteria are trapped in the channels.	Chip pre‐treatment: 30 min in water to saturate and thus reduce evaporation.
Bacteria in prepared sample [CFU/ml]	<250	≈1.5 × 10^8^	5 × 10^5^	10^7^	>10^4^	10^7^
Turnaround time (ex. preculture) [h]	5–10 (5–10)	27.8–33.8 (9.8)	30.1–36.1 (12.1)	13–19 (≈1)	>2.5 (>0.5)	13–15 (1–3)
Challenges remaining						
Demonstrated for complex samples	Yes	Yes	Yes	No	No	No
Hands‐on time	Low	average	average	Low	High	Low
Demonstrated for several pathogens	Yes	Yes	Yes	No	Yes (mainly for *E. coli*)	Yes
Sophisticated equipment required / Point of care possible	No / Yes	Yes / No	Yes / No	No / Yes	Yes / No	No / Yes
Scalability for high throughput applications	Good	Good	Good	Good	Poor	Good
Method‐specific challenges		Long preculture	Long preculture	Evaporation restricts longer measurements	Channel size has to match bacteria: high variability results for *K. pneumonia* and *S. saprophyticus*	

## 4 Experimental Section

Two Screen printed electrodes (SPEs) were preevaluated for suitability as application in the 3D printed device. The electrodes were purchased from PalmSens (PalmSens BV, Houten, Netherlands). First an ItalSens IS‐C SPE with graphite working and counter electrode, and a silver pseudo reference electrode, and second a BVT‐AC1 with gold working and counter electrode with a Ag/AgCl reference electrode. LB medium (Lennox) and LB‐Agar (Luria/Miller) were purchased from Carl Roth (Carl Roth GmbH + Co. KG, Karlsruhe, Germany). Fetal calf serum (FCS), oxytetracycline hydrochloride, and kanamycin sulfate were purchased from Sigma Aldrich (Sigma‐Aldrich Chemie GmbH, Taufkirchen, Germany). Resazurin was purchased from VWR (VWR International GmbH, Darmstadt, Germany). A Minitron (Infors HT, Bottmingen, Switzerland) was used as an incubator.

### Device Fabrication

Prototype devices were fabricated as previously described.^[^ ^42^ ^]^ Briefly, a CAD model was created in Autodesk Fusion 360 (Autodesk, United States) and processed in the slicer software Ultimaker Cura (Version 4.6.1, Ultimaker, Netherlands). Prototype devices were printed with an Ultimaker 3 (Ultimaker, Netherlands) FDM 3D printer with a 0.4 mm nozzle head using the polymer PMMA‐transparent (PMM300XCLR) purchased from filamentworld.com (Germany). As general parameters, a printing temperature of 245 °C, printing speed of 70 mm s^−1^, layer height of 0.1 mm, and fan speed at 50% was used. The printing process was paused at the appropriate layer (modification of the G‐code by post‐processing scripts available by default) to insert the SPEs and continued afterward. As SPEs, commercially available ItalSens IS‐C SPEs were used for the antibiotic susceptibility test experiments. To ensure a firm bond between the electrode strip backing material and the already solidified 3D print, a small drop of superglue was applied before insertion of the electrode, ensuring that the glue adhered only to the bottom of the electrode backing material. After fabrication, the devices were sterilized under a sterile work bench with UV light for 30 min from each side and closed with a sterile luer‐lock cap.

### Bacteria Culture

Cultures of *Escherichia coli* (DSM 498), multidrug‐resistant *Staphylococcus aureus* (MRSA, DSM 28766), susceptible *S. aureus* (DSM 799), *Klebsiella pneumoniae* (DSM 103706), *K. pneumoniae* (DSM 30104), *Pseudomonas aeruginosa* (DSM 102273), and *P. aeruginosa* (DSM 25123) were cultured overnight in LB medium at 37 °C and 100 rpm before being used to prepare spiked serum samples. For each experiment, a new cryo‐vial was thawed and cultured. Strains were purchased from the Leibniz Institute DSMZ‐Deutsche Sammlung von Mikroorganismen und Zellkulturen GmbH (Braunschweig, Germany) and stored at −80 °C in glycerol stocks.

### Antibiotic Susceptibility Test by Agar Diffusion Assay

The agar diffusion assay was performed for the tested bacterial strains with the antibiotics kanamycin and oxytetracycline in order to confirm the resistances listed in the online library bacdive.^[^ ^34^ ^]^ A bacterial solution with an OD_600_ of 0.025 in LB media was created from an overnight culture and 100 µl was evenly distributed on a standard microbial petridish with LB agar. Sterile filter paper discs were soaked with 10 µl (1 mg ml^−1^) antibiotic solution in PBS, resulting in 10 µg antibiotic per disc and placed on agar ≈5 min after the innoculation. After 15 min, the petridishs were turned upside down and placed in an incubator with 37 °C. After 24 h the plates were analyzed and pictures of the inhibition zones around the antibiotic soaked discs were taken.

### Antibiotic Susceptibility Test – Differential Pulsed Voltammetry

**Figure 6:**
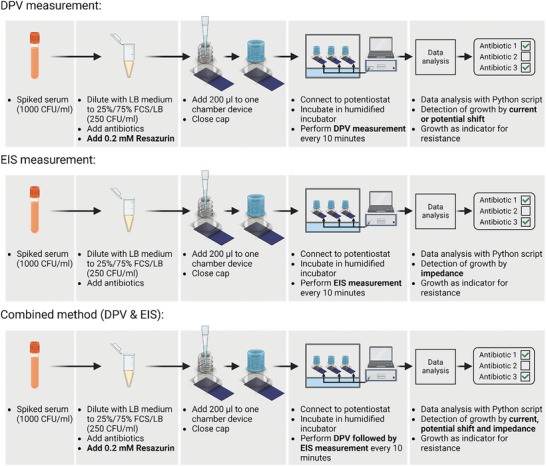
Workflow for the electrochemical AST method with the 3D printed prototype. Workflows from spiked serum are shown for combined method (DPV & EIS) and for performing as separate measurements. Differences between workflows are highlighted in bold letters.

Resazurin concentration, which decreased as a result of active metabolism, was measured by the electrochemical method of DPV, as illustrated in **Figure** **6**. Sterile 3D printed prototypes were prepared with IS‐C SPEs, as previous described. Serum was spiked with an overnight bacterial culture to 1000 CFU/ml, corresponding to a bloodstream infection of 1000 CFU/ml, and diluted with LB medium to 25%/75% FCS/LB (results in 250 CFU/ml). After 0.2 mm Resazurin and the antibiotic were added, 200 µl were pipetted into the device (t = 0 h). The device was closed and the sensor was connected to the potentiostat PalmSens 4. The connected device was then incubated in a humidified incubator at 37 °C without shaking and the amount of metabolized resazurin was evaluated with DPV (*E*_begin_ = ‐0.8 V; *E*_end_ = 0.0 V; *E*_pulse_ = 0.05 V; *t*_pulse_ = 0.01 s; Scan rate = 0,05 V/s) every 10 min over 20 h. Measurements were performed with the PalmSens4 (PalmSens BV, Houten, Netherlands) potentiostat and the Software PSTrace (Version 5.9.2317, PalmSens BV, Netherlands).

### Antibiotic Susceptibility Test – Electrochemical Impedance Spectroscopy

The experimental setup for EIS measurement (Figure 6) was similar to the one described above for DPV measurements, except that resazurin was not required. Instead of the DPV protocol, the EIS measurement protocol at frequencies of 10^5^, 10^4^, 10^3^, 10^2^, 10, and 1 Hz with an *E*_dc_  =  0.3 V and *E*_ac_ = 0.01 V was applied using the PalmSens4 and the Software PSTrace.

### Antibiotic Susceptibility Test – Combined Method (DPV & EIS)

For the combined method, both measurements (DPV & EIS) were necessary. They did not require a separate experimental setup and can thereby be performed in parallel on one device. The initial preparation (Figure 6) was identical to the one for the DPV measurement, only the measurement differs. The measurements for DPV and EIS were performed sequentially with the parameters described above, as the protocol for DPV was performed first and after a 10 s pause the protocol for EIS was performed.

### Bioinformatic & Statistical Analysis

Electrochemical data were recorded using PSTrace and exported for further analysis using a Python script (version 3.11.3). The three most promising parameters were used for bioinformatics analysis. For the DPV measurement, these were first, the current (*I*) at −0.3 V, which was related to the resazurin concentration, and second, the potential (*U*) of the peak maximum, which shifted to a more positive potential as pH decreased. The third parameter was the impedance (*Z*) at 100 kHz of the EIS measurement. The detection of bacterial growth in the acquired time series was performed by change point detection (Pruned Exact Linear Time (PELT) algorithm) using the ruptures package version 1.1.7 ^[^ ^43^ ^]^ and significance detection by the SciPy package version 1.10.1.^[^ ^44^ ^]^ Potential values for detecting the Peakshift were used without prior adjustments. For *I* and *Z*, only values after reaching equilibrium, determined according to Dalheim et al.,^[^ ^45^ ^]^ were used. In addition, the change point detection method for detecting growth by current was performed on the first derivative of *I*. Each change point was then analyzed for significant differences between the first derivative of the time series values used for change point detection and the corresponding control without bacterial growth. Only change points with significant differences (paired *t*‐test, *p* < 0.05 was considered as significant) to 4 out of 5 independent control samples were considered as significant change points and displayed in plots as growth detected. Evaluation of a combination of parameters was performed by calculating a score ranging from 0 to 3, which increased by 1 for each significant change point of a parameter at the analysis timepoint and 1 h before. The score was restricted to a maximum increase of 1 by each parameter, with a score of 2 or more indicating growth detected. Plots were created using the package matplotlib.

The performance of the AST method compared to the AST method using the agar diffusion test was calculated according to Equations (2)–(5), whereby the presence of a resistance to the tested antibiotic was defined as a positive condition. The number of positive (P) and negative (N) conditions were determined according to the results of the agar diffusion test. True positives (TP) and true negatives (TN) were test results of the AST method to be evaluated that indicate the presence or absence of resistance in accordance with the agar diffusion test. False positive (FP) and false negative (FN), on the other hand, were test results of the AST method to be evaluated that indicated the presence or absence of resistance contrary to the results of the agar diffusion test.

(2) $$\mathrm{Sensitivity}\;=\frac{TP}{T}\;$$

(3) $$\mathrm{Specificity}\;=\frac{TN}{N}\;$$

(4) $$\mathrm{Positive}\;\mathrm{predictive}\;\mathrm{value}\;\left(\mathrm{PPV}\right)\;=\frac{TP}{\mathrm{TP}+\mathrm{FP}}\;$$

(5) $$\mathrm{Negative}\;\mathrm{predictive}\;\mathrm{value}\;\left(\mathrm{NPV}\right)\;=\frac{TN}{TN+FN}\;$$

## Conflict of Interest

O.R. and H.P.D. are inventors on a patent application (DPMA Deutsches Patent‐ und Markenamt DRN: 2023082213530700DE) that covers the electrochemical AST system.

## Author Contributions

O.R., L.K., S.L., and H.P.D. conceptualized the idea of the study. O.R. designed methodology; contributed to software development; and performed formal analysis, investigation, data curation, and visualization. L.K. and H.P.D. performed funding acquisition. S.L. and H.P.D. performed supervision. O.R. and H.P.D. wrote original draft. O.R., L.K., S.L., and H.P.D. wrote, reviewed, and edited the final manuscript.

## Supporting information

Supporting Information

## Data Availability

The data that support the findings of this study are available from the corresponding author upon reasonable request.
