# Supplementary material for: Rapid Phenotypic Antibiotics Susceptibility Analysis by a 3D Printed Prototype
Source: Adv Sci (Weinh). 2024 Mar 26;11(21):2308806. doi: 10.1002/advs.202308806 (PMC11151047; doi:10.1002/advs.202308806)
Supplement: Supplementary file 1 — Supporting Information [file ADVS-11-2308806-s001.pdf]

ADVANCED SCIENCE
Open Access

Supporting Information

for *Adv. Sci.*, DOI 10.1002/advs.202308806

Rapid Phenotypic Antibiotics Susceptibility Analysis by a 3D Printed Prototype

*Oliver Riester, Lars Kaiser, Stefan Laufer and Hans-Peter Deigner**

Supporting Information

Rapid Phenotypic Antibiotics Susceptibility Analysis by a 3D Printed Prototype

*Oliver Riester, Lars Kaiser, Stefan Laufer, Hans-Peter Deigner **

* Corresponding author. Email: deigner@gmx.de

Abstract

One of the most important public health concerns is the increase in antibiotic-resistant pathogens and corresponding treatment of associated infections. Addressing this challenge requires more efficient use of antibiotics, achievable by the use of evidence-based, effective antibiotics identified by antibiotic susceptibility testing (AST). However, the current standard method of phenotypic AST used for this purpose requires 48 hours or more from sample collection to result. Until results are available, broad-spectrum antibiotics are used to avoid delaying treatment. The turnaround time must therefore be shortened in order for the results to be available before the second administration of antibiotics. The phenotypic electrochemical AST method presented here identifies effective antibiotics within 5 - 10 hours after sampling. Spiked serum samples, including polymicrobial samples, with clinically relevant pathogens and respective concentrations commonly found in bloodstream infections (*E. coli*, *S. aureus*, *K. pneumoniae and P. aeruginosa*) are used. Direct loading of the test with diluted serum eliminates the need for a pre-culture, as required by existing methods. Furthermore, by combining several electrochemical measurement procedures with computational analysis, allowing the method to be used both online and offline, the AST achieves a sensitivity of 94.44% and a specificity of 95.83% considering each replicate individually.

Keywords: antibiotic susceptibility testing, phenotypic, antibiotic resistances, 3D printer, screen printed electrodes, no preculture

Experimental Section

Differential Pulse Voltammetry (DPV) for Concentration Determination of Resazurin

Measurement of resazurin by the DPV method was investigated with two SPEs for linearity and signal strength. Electrodes were electrochemically cleaned before use by 5 cycles of cyclic voltammetry in PBS. Solutions with 0; 0.01; 0.05; 0.1; 0.5 and 1 mM Resazurin solved in PBS, 25/75% FCS/LB medium or 100% FCS were tested. Measurements were performed with the PalmSens4 potentiostat and the Software PSTrace after equilibrium was reached. The following parameters were used for DPV measurement: IS-C electrode (E_{begin} = -0.8 V; E_{end} = 0.0 V; E_{pulse} = 0.05 V; t_{pulse} = 0.01 s; Scan rate = 0,05 V/s) and BVT-AC1 electrode (E_{begin} = -1.4 V; E_{end} = 0 V; E_{pulse} = 0.5 V; t_{pulse} = 0.01 s; Scan rate = 0,05 V/s). DPV curves were exported from PSTrace after moving average baseline correction and plotted using GraphPad Prism 8 (GraphPad Software, USA).

Pre-wetting of Electrodes

Devices were pre-treated to evaluate the influence of wetting effects on the initial time to reach an equilibrium state. Therefore, sterile devices (aerobic design, one chamber) were loaded with 100 µl millipore water, PBS, LB-medium, 75% LB 25% FCS, 100% FCS or none (untreated) and incubated at 37°C in a humidified incubator for 2 h. Afterwards, the added liquid was removed and the devices were loaded with 200 µl sample consisting of 50 µl FCS and 150 µl LB medium with 0.2 mM Resazurin. For this test samples were used without bacteria and antibiotics as these have shown no effect on the initial phase of the measurement so far.

Polymicrobial Samples

The suitability of the electrochemical prototype for evaluating efficacy of tested antibiotics for polymicrobial samples was tested for combinations of three strains spiked (each strain to 333 CFU/ml) in human plasma from healthy donors purchased from Sigma-Aldrich (Sigma-Aldrich Chemie GmbH, Taufkirchen, Germany). Strain combinations were selected from the 7 strains to obtain 4 combinations ranging from 3 sensitive (S) to 3 resistant (R) strains (SSS, RSS, RRS, RRR) per antibiotic to be tested. Each polymicrobial sample was mixed in a labeled 1.5 ml reaction tube by adding 180 µl LB-medium, 5 µl of 10 mM Resazurin stock, 2.5 µl of the respective 10 mg/ml antibiotic stock and 62.5 µl spiked human plasma (each strain 333 CFU/ml; combined around 1000 CFU/ml). Then, 200 µl of samples were transferred to electrochemical devices (aerobic design, one chamber), connected to the potentiostat PalmSens4 and incubated in a humidified incubator at 37°C. DPV and EIS measurements were performed every 10 minutes for 20 h and growth was detected using the combined method approach.

Minimum Inhibitory Concentration Assay

Minimum inhibitory concentration (MIC) was tested according to an adapted version of Kowalska-Krochmal et al.[1] and ISO 20776-1.[2] The assay was performed as broth microdilution in a 96-wellplate (TPP Techno Plastic Products AG, Trasadingen, Switzerland) design and on the electrochemical devices to evaluate comparability for MIC measurements with the prototype using human serum samples from healthy donors purchased from Sigma-Aldrich (Sigma-Aldrich Chemie GmbH, Taufkirchen, Germany) spiked with corresponding bacteria of an overnight culture to match a clinical blood sample. The sensitive strains *S. aureus* (DSM 799) and *K. pneumoniae* (DSM 30104) were choosen as example strains based on their relevance for bloodstream infections (BSI) and their sensitive character to the test antibiotics kanamycin and oxytetracycline.

For comparability reasons, both MIC assays (broth microdilution and prototype) were performed in an overall volume of 200 µl 25% human serum 75% LB-medium. For the broth microdilution method a 96-wellplate was loaded with 50 µl of a 10-fold dilution series (400 µg/ml – 0.4 µg/ml corresponding to 100 µg/ml – 0.1 µg/ml in endvolume of 200 µl) of kanamycin or oxytetracycline in LB-medium. Then, 50 µl human plasma spiked with 2 x 10^6 CFU/ml (final concentration in 200 µl according to Kowalska-Krochmal et al.:[1] 5 x 10^5 CFU/ml) and 100 µl LB-medium were added, resulting in 200 µl per well. The lid of the wellplate was closed, sealed with parafilm and incubated for 24 h at 37°C in an incubator. After 24 h photos were taken and growth was visually analyzed.

The MIC assay in the electrochemical prototype was performed similarly with some modifications: 250 µl were prepared in 1.5 ml reaction tubes, of which 200 µl were added to the device. 62.5 µl of a 10-fold dilution series (400 µg/ml – 0.4 µg/ml corresponding to 100 µg/ml – 0.1 µg/ml in endvolume of 250 µl) of kanamycin or oxytetracycline in LB-medium were added to labeled 1.5 ml reaction tubes. Then, 62.5 µl human plasma spiked with 1000 CFU/ml and 125 µl LB-medium containing 0.4 mM Resazurin (corresponds to 0.2 mM Resazurin in 250 µl) was added, resulting in 250 µl per tube. The sample was mixed and 200 µl were loaded into an electrochemical device (aerobic design, one chamber), connected to the potentiostat PalmSens4 and incubated in a humidified incubator at 37°C without shaking. DPV and EIS measurements were performed every 10 minutes for 20 h and growth was detected using the combined method approach.

Results and Discussion

DPV - Signal Changes

The measurement of resazurin metabolism by DPV was indicated by a change in current flow at the peak characteristic of resazurin. An increase in bacterial growth was usually accompanied by a decrease in current intensity (e.g. Figure S2 to S8). In the case of *K. pneumoniae*, however, in addition to the decrease, a time-shifted increase was also observed, which also occurred in the case of *P. aeruginosa*, but without the characteristic decrease. As this behavior was specific for these pathogens, it was tested whether it also occurs without the addition of resazurin, which occurred to be the case, as shown in Figure S11, although to a lesser extent. As the signal is not entirely related to resazurin, it is assumed, that it originates from a metabolic product. Nevertheless, a detailed evaluation of the underlying mechanisms has not yet been carried out. For the measurement of growth, however, the use of this effect is also suitable, since it only occurs when growth is present.

Antibiotic Susceptibility Test by Bacterial Metabolism of Resazurin – Linearity by Differential Pulsed Voltammetry (DPV)

Several concentrations of resazurin in PBS or 25/75% FCS/LB were analyzed by DPV. The results obtained are shown in Figure S9 for IS-C and BVT screen printed electrodes (SPEs). Measurements in PBS showed nonlinear behavior for IS-C but not for BVT SPEs, as shown in Figure S10, which is likely caused by diffusion limitations at the electrode surface, since the carbon-based (IS-C) SPEs have a larger surface area than the tested gold electrodes (BVT) due to the porous structures of the electrode material.[3,4] However, when tested in 25/75% FCS/LB, the IS-C electrodes also showed a linear behavior, probably due to the biomolecules present in the FCS and LB media, which blocked part of the electrochemically available surfaces and reduced the reaction rate, making diffusion limitation less crucial. A decrease in the reaction rate of resazurin in PBS to 25/75% FCS/LB is also evident when comparing the maximum current values for the same resazurin concentration, e.g., 52.13 ± 4.80 µA in PBS versus 21.37 ± 6.31 µA in 25/75% FCS/LB for 0.5 mM resazurin at the IS-C electrode. This trend continued in 100% FCS (Figure S10) to 4.093 µA for 0.5 mM resazurin. As shown in Figure S10, DPV measurements were possible in 100% FCS, but 25/75% FCS/LB was chosen in the antibiotic susceptibility testing (AST) experiments since the parallel EIS measurements in 100% FCS led to non-reproducible results and interference.

Effect of Pre-wetting Electrodes on Time to Initial Equilibrium

The effect of pre-treatment of the electrodes by wetting prior to the experiment was assessed by the time until a stable current ($I_{\text{initial increase}}$) ,according to Dalheim et al.,[5] and the time from which a steady rise in the measured impedance from the minimum (Z_{low}) was observed. The initial increase in I, which was observed in all measurements with and without bacteria and was accompanied by an initial decrease in Z, was shortened by the pre-treatment of the electrode. However, different effects were observed depending on the medium used. As shown in Figure S12, using millipore water for pre-treatment showed no effect ($I_{\text{initial increase}}$: 3h10min; Z_{low}: 0h20min – 1h40mi) compared to the untreated device ($I_{\text{initial increase}}$: 3h30min; Z_{low}: 0h20min – 1h40min), whereas PBS led to a reduction in the time to reach a stable current by more than one hour, but did not reduce time until impedance increased ($I_{\text{initial increase}}$: 2h20min; Z_{low}: 0h30min – 1h50min). The use of LB medium to pre-wet the electrodes showed a similar trend as PBS but to a greater extent, as the equilibrium of I was observed after 30 min, but was unstable as it dropped rapidly thereafter. In addition, no change was observed for Z_{low} (0h10min - 1h50min) compared to the untreated device. Pre-treatment with 75% LB 25% FCS showed better results, although $I_{\text{initial increase}}$ was reached later, the current was more stable afterwards and Z increased

from its low value after only 1 hour and 10 minutes ($I_{initial\ increase}$: 2h00min; Z_{low}: 0h10min - 1h10min), shortening the initial equilibrium phase to about 2 hours. The same was observed for the pre-treatment with 100% FCS ($I_{initial\ increase}$: 2h00min; Z_{low}: 0h20min – 1h10min), whereas pre-treatment with FCS also showed a permanent drop in the measured current to around 4 µA, while values between 6 and 5 µA were observed for the untreated device.

Polymicrobial Samples

The method was further tested for samples containing multiple strains in order to demonstrate its suitability for assessing antibiotic susceptibility for polymicrobial infections directly from the patient sample without prior isolation of the strains. The pathogen combinations for the tests with 3 resistant pathogens differ, as only 2 of the tested strains were resistant to oxytetracycline (see Figure 3). Therefore, instead of *S. aureus* (DSM 28766), which was only resistant to kanamycin, *P. aeruginosa* (DSM 25123) was added as a third strain for treatment with oxytetracycline, as it showed tolerance to oxytetracycline, although not complete resistance. The method presented, determines whether growth is present or not, i.e. it can determine whether one or more strains present in the sample are resistant to the treatment (growth is detected) or whether all strains present are susceptible (no growth is detected). For clinical decision-making an antibiotic effective against all bacteria present in the sample is then selected.

The results presented in Figure S13 show good agreement between the expected growth when one or more of the three pathogens are resistant and the growth observed with the combined method. The exception concerned treatments with kanamycin when 1 or 2 resistant pathogens were present. Contrary to expectations, no growth was observed in the test with these combinations. Two hypotheses were tested: firstly, as kanamycin has already been shown to slow the growth of *K. pneumoniae* (DSM103706) (see Figure S5), it was hypothesised that sufficient growth would only be present after 20 hours and, therefore, was not detected. However, no growth was visually observed in the device even after 48 hours of incubation. The second hypothesis was that the resistance mechanism is dependent on the bacterial concentration, e.g. in the case of enzymatic inactivation of the aminoglycoside kanamycin.[6] Since the initial concentration of bacteria in the test was only around 333 CFU/ml per strain (1000 CFU/ml in total), compared to 1000 CFU/ml in the experiments where resistance (growth) was detected (see Figure S5), the experiment was repeated for these combinations with 1000 CFU/ml per strain (3000 CFU/ml in total Growth was observed at the higher initial concentrations of bacteria (see Figure S14), indicating that the resistance mechanism of the pathogens depends on the amount of bacteria present.

Minimum Inhibitory Concentration (MIC)

A combination of two sensitive strains were randomly selected from the strains used in the study and the MIC for these was evaluated using both standard broth microdilution and 3D printed prototype with electrochemical detection by the combined method. The results are shown in Figure S15 and demonstrate agreement between the broth microdilution test adapted from ISO 20776-1 [2] and the combined method in the 3D printed prototype.

Table S1. Overview of AST results by measurement methods for treatment without antibiotic (None), with Kanamycin (K) or with Oxytetracycline (O). Sensitivity, specificity, PPV and NPV were calculated for each replicate in comparison to the result of the agar diffusion assay.

Bacterial Strain	Treat-ment	Resistance		Current @ - 0.3 V (DPV)		Potential of current maximum (DPV)		Impedance @ 100 kHz (EIS)		Combination of Current, Potential and Impedance (Score)	
		BacDive Library	Agar diffusion	growth detected (No.of repl.)	Detection time / h	growth detected (No.of repl.)	Detection time / h	growth detected (No.of repl.)	Detection time / h	growth detected (No.of repl.)	Detection time / h
E. coli (DSM 498)	None	N / A	N / A	3 / 3	4.89 ± 0.21	3 / 3	5.56 ± 0.44	3 / 3	6.5 ± 0.14	3 / 3	5.06 ± 0.08
	K	-	-	1 / 3	5.83	0 / 3	N / A	1 / 3	13.5	0 / 3	N / A
	O	-	-	2 / 3	6.75 ± 1.42	0 / 3	N / A	0 / 3	N / A	0 / 3	N / A
S. aureus (DSM 28766)	None	N / A	N / A	3 / 3	7.67 ± 1.53	3 / 3	10.05 ± 0.57	3 / 3	8.11 ± 0.48	3 / 3	7.72 ± 0.75
	K	+	+	3 / 3	6.56 ± 0.44	3 / 3	10.39 ± 0.67	3 / 3	8.28 ± 0.34	3 / 3	7.06 ± 0.83
	O	-	-	0 / 3	N / A	0 / 3	N / A	1 / 3	18.67	0 / 3	N / A
S. aureus (DSM 799)	None	N / A	N / A	3 / 3	7.06 ± 0.96	3 / 3	8.17 ± 0.36	3 / 3	6.45 ± 0.91	3 / 3	5.33 ± 0.72
	K	-	-	0 / 3	N / A	0 / 3	N / A	0 / 3	N / A	0 / 3	N / A
	O	-	-	1 / 3	13.17	0 / 3	N / A	2 / 3	16.84 ± 1.34	0 / 3	N / A
K. pneumonia (DSM 103706)	None	N / A	N / A	3 / 3	5 ± 0.36	3 / 3	5.28 ± 0.21	3 / 3	5.44 ± 0.08	3 / 3	4.94 ± 0.28
	K	+	+	3 / 3	8 ± 0.94	3 / 3	9.5 ± 0.98	3 / 3	10.11 ± 1.1	3 / 3	9.28 ± 0.88
	O	+	+	3 / 3	7.11 ± 0.16	3 / 3	5.17 ± 0.41	3 / 3	5.5 ± 0	3 / 3	5.11 ± 0.16
K. pneumonia (DSM 30104)	None	N / A	N / A	3 / 3	5.67 ± 0.62	3 / 3	6.67 ± 0.62	3 / 3	6.83 ± 0.27	3 / 3	6.11 ± 0.16
	K	-	-	2 / 3	5.17 ± 0.5	0 / 3	N / A	3 / 3	13.39 ± 6.05	1 / 3	4.33
	O	-	-	0 / 3	N / A	0 / 3	N / A	2 / 3	15.67 ± 1.67	0 / 3	N / A
P. aeruginosa (DSM 102273)	None	N / A	N / A	2 / 3	9.25 ± 0.58	0 / 3	N / A	1 / 3	11.67	3 / 3	7.72 ± 0.52
	K	-	-	2 / 3	8.17 ± 0.5	0 / 3	N / A	2 / 3	9.42 ± 0.59	2 / 3	7.58 ± 1.25
	O	-	+	1 / 3	9	1 / 3	7.83	1 / 3	18.5	3 / 3	8.61 ± 0.87
P. aeruginosa (DSM 25123)	None	N / A	N / A	3 / 3	6.56 ± 0.28	1 / 3	18.5	3 / 3	7.84 ± 0.24	3 / 3	7.22 ± 0.28
	K	-	-	0 / 3	N / A	0 / 3	N / A	0 / 3	N / A	0 / 3	N / A
	O	-	+	3 / 3	8.55 ± 0.21	1 / 3	8.17	3 / 3	11.17 ± 1.47	3 / 3	10.22 ± 1.59
Sensitivity | Specificity				83.33 % | 75 %		61.11 % | 100 %		83.33% | 62.5 %		94.44 % | 95.83 %	
PPV | NPV				71.43 % | 85.71 %		100 % | 77.42 %		62.5 % | 83.33 %		94.44 % | 95.83 %	

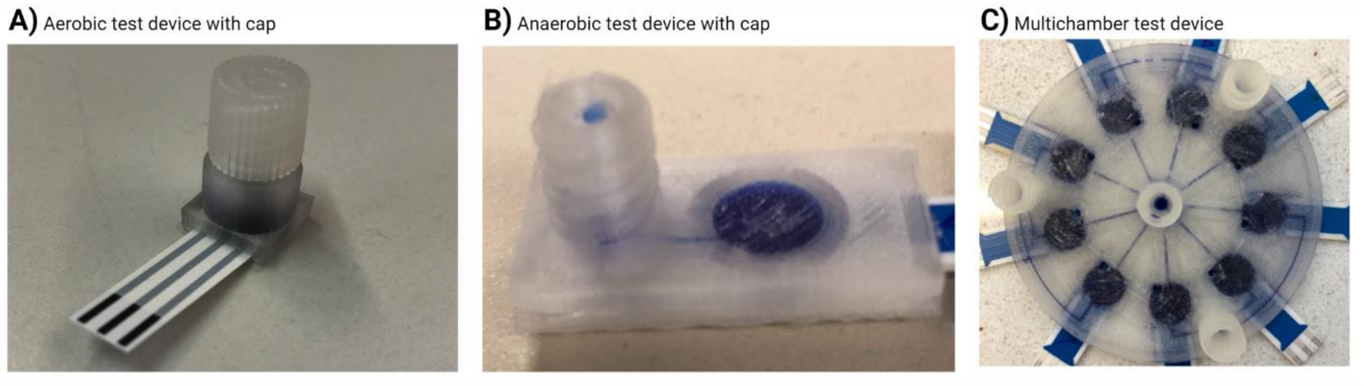

Figure S1. Pictures of possible 3D printed device designs. Devices with implemented SPEs (A: IS-C; B, C: BVT) filled with 25/75% FCS/LB containing 1 mM Resazurin. A) Aerobic design with air space for oxygen exchange and Luer-Lock cap for sterile sealing. B) Anaerobic design without air space for oxygen exchange and Luer lock closure for sterile sealing. C) Multi-chamber variant of the anaerobic design with Luer-Lock closures (inlet and outlet) for sterile sealing. Devices were printed from PMMA (very low oxygen permeability).

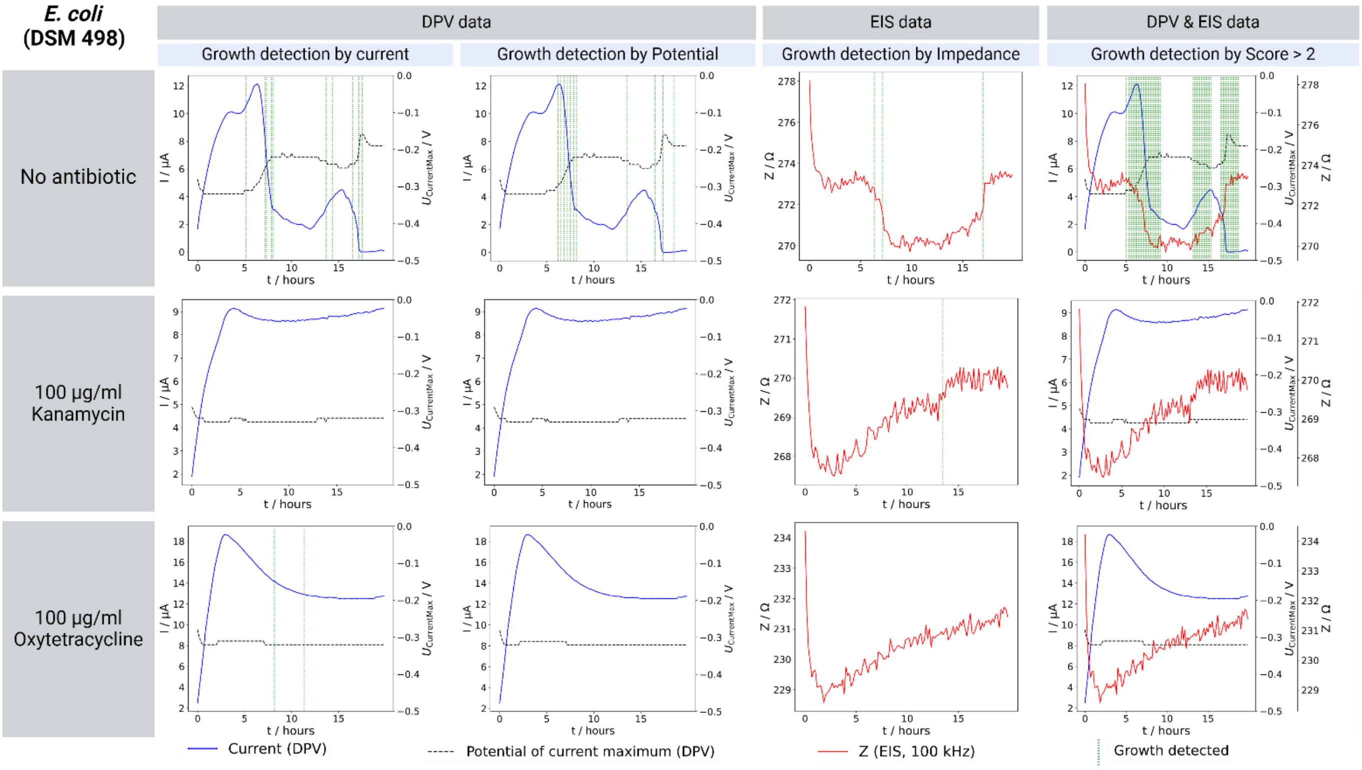

Figure S2. Electrochemical measurement and bioinformatics analysis for antibiotic susceptibility of *Escherichia coli* (DSM 498). Bacterial growth of *E. coli* with 100 µg/ml kanamycin, 100 µg/ml oxytetracycline, or without antibiotic was measured by differential pulsed voltammetry (DPV) and electrochemical impedance spectroscopy (EIS) in aerobic test device with an IS-C screen printed electrode. The plots show one of three representative replicates with evidence of growth by different parameters (current (*I*), potential (*U*), impedance (*Z*) and combined method (score)).

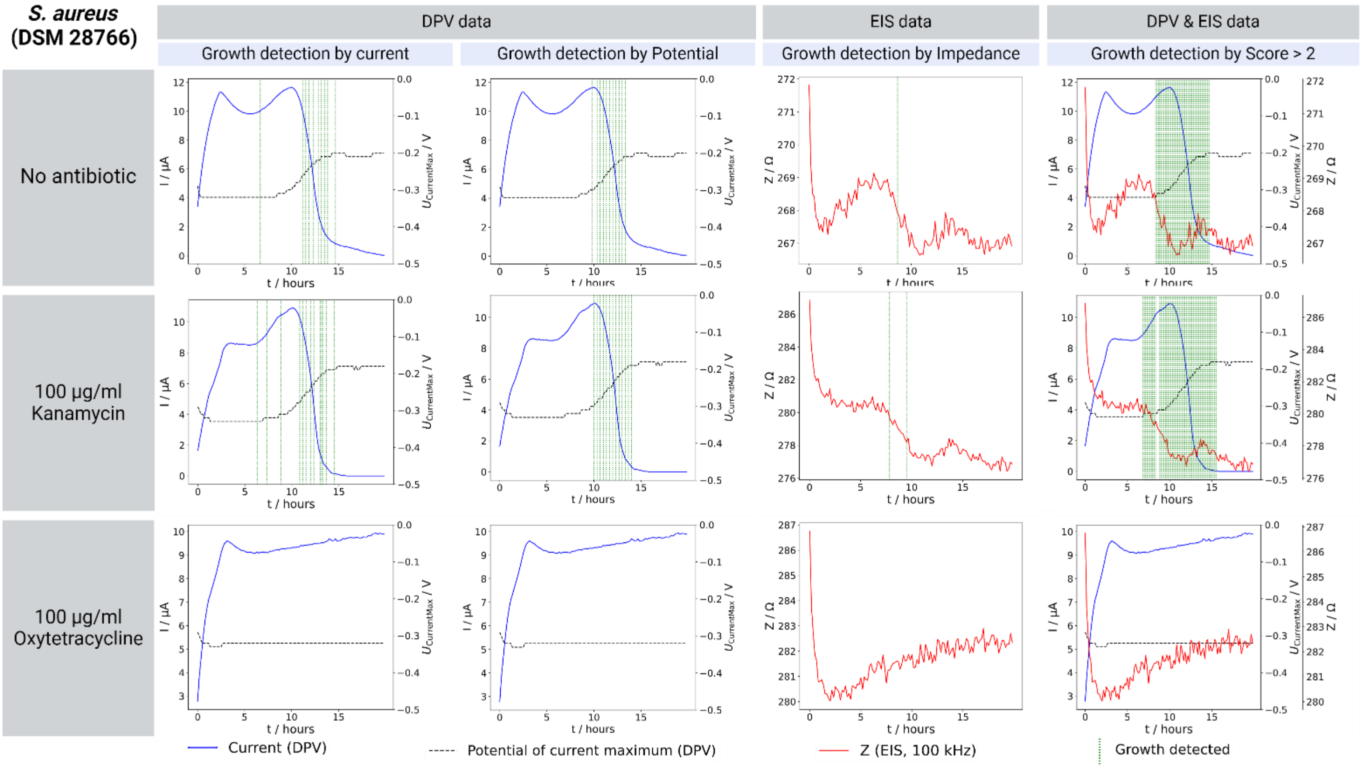

Figure S3. Electrochemical measurement and bioinformatics analysis for antibiotic susceptibility of *Staphylococcus aureus* (DSM 28766). Bacterial growth of *S. aureus* with 100 µg/ml kanamycin, 100 µg/ml oxytetracycline, or without antibiotic was measured by differential pulsed voltammetry (DPV) and electrochemical impedance spectroscopy (EIS) in aerobic test device with an IS-C screen printed electrode. The plots show one of three representative replicates with evidence of growth by different parameters (current (*I*), potential (*U*), impedance (*Z*) and combined method (score)).

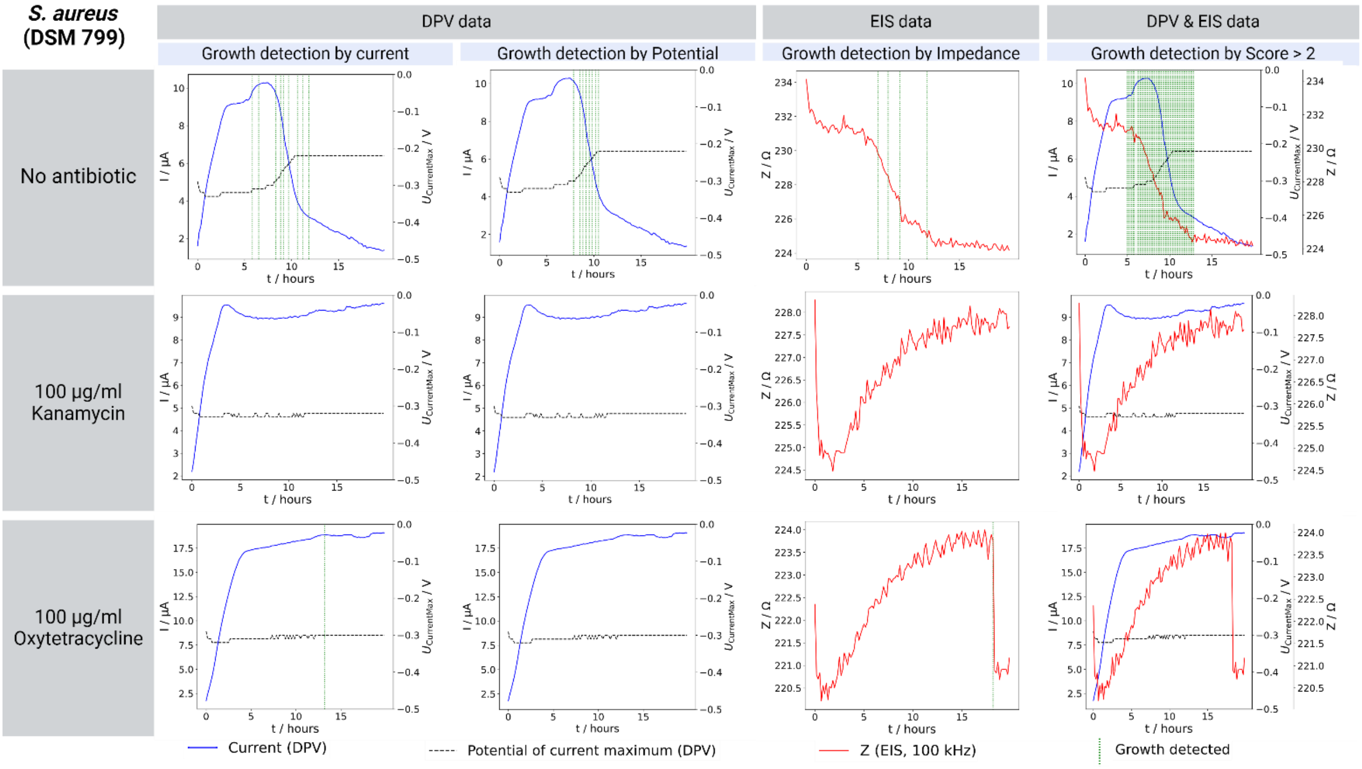

Figure S4. Electrochemical measurement and bioinformatics analysis for antibiotic susceptibility of *Staphylococcus aureus* (DSM 799). Bacterial growth of *S. aureus* with 100 µg/ml kanamycin, 100 µg/ml oxytetracycline, or without antibiotic was measured by differential pulsed voltammetry (DPV) and electrochemical impedance spectroscopy (EIS) in aerobic test device with an IS-C screen printed electrode. The plots show one of three representative replicates with evidence of growth by different parameters (current (*I*), potential (*U*), impedance (*Z*) and combined method (score)).

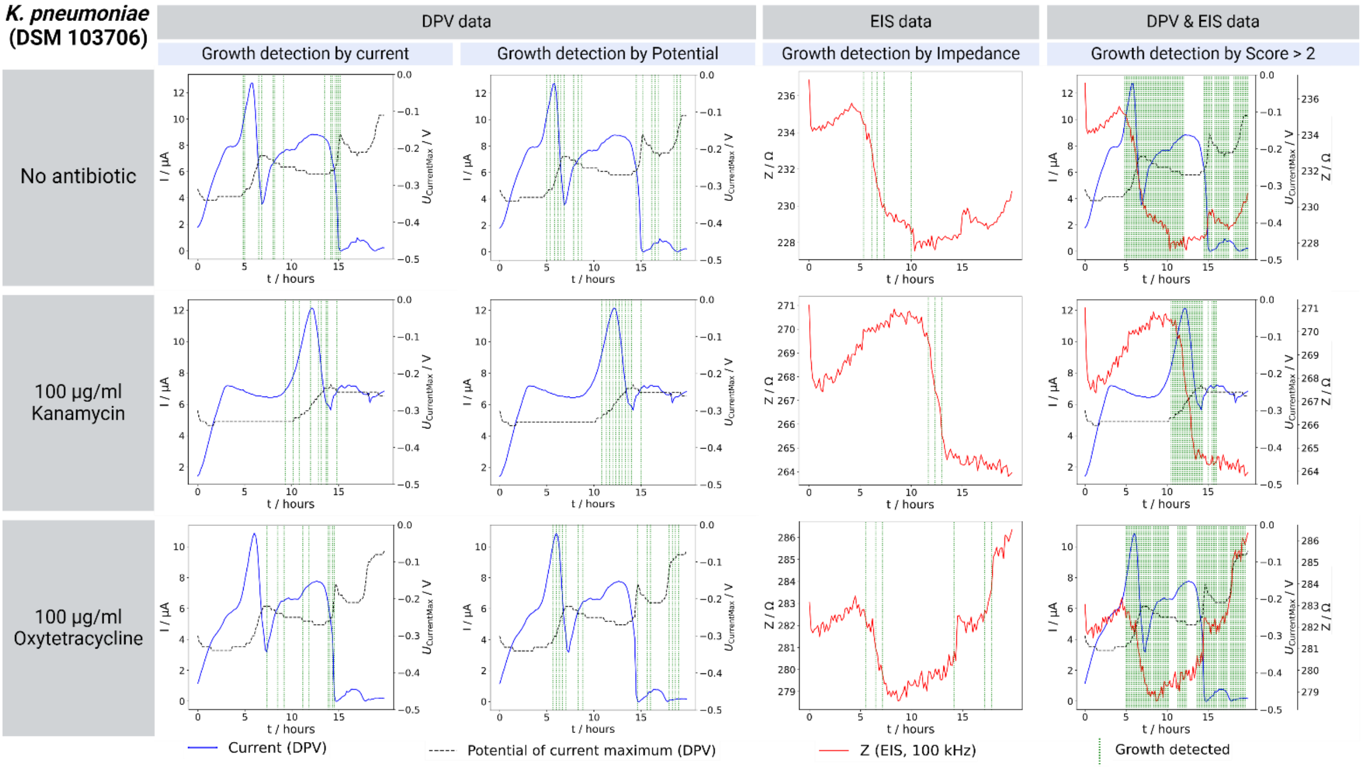

Figure S5. Electrochemical measurement and bioinformatics analysis for antibiotic susceptibility of *Klebsiella pneumoniae* (DSM 103706). Bacterial growth of *K. pneumoniae* with 100 µg/ml kanamycin, 100 µg/ml oxytetracycline, or without antibiotic was measured by differential pulsed voltammetry (DPV) and electrochemical impedance spectroscopy (EIS) in aerobic test device with an IS-C screen printed electrode. The plots show one of three representative replicates with evidence of growth by different parameters (current (*I*), potential (*U*), impedance (*Z*) and combined method (score)).

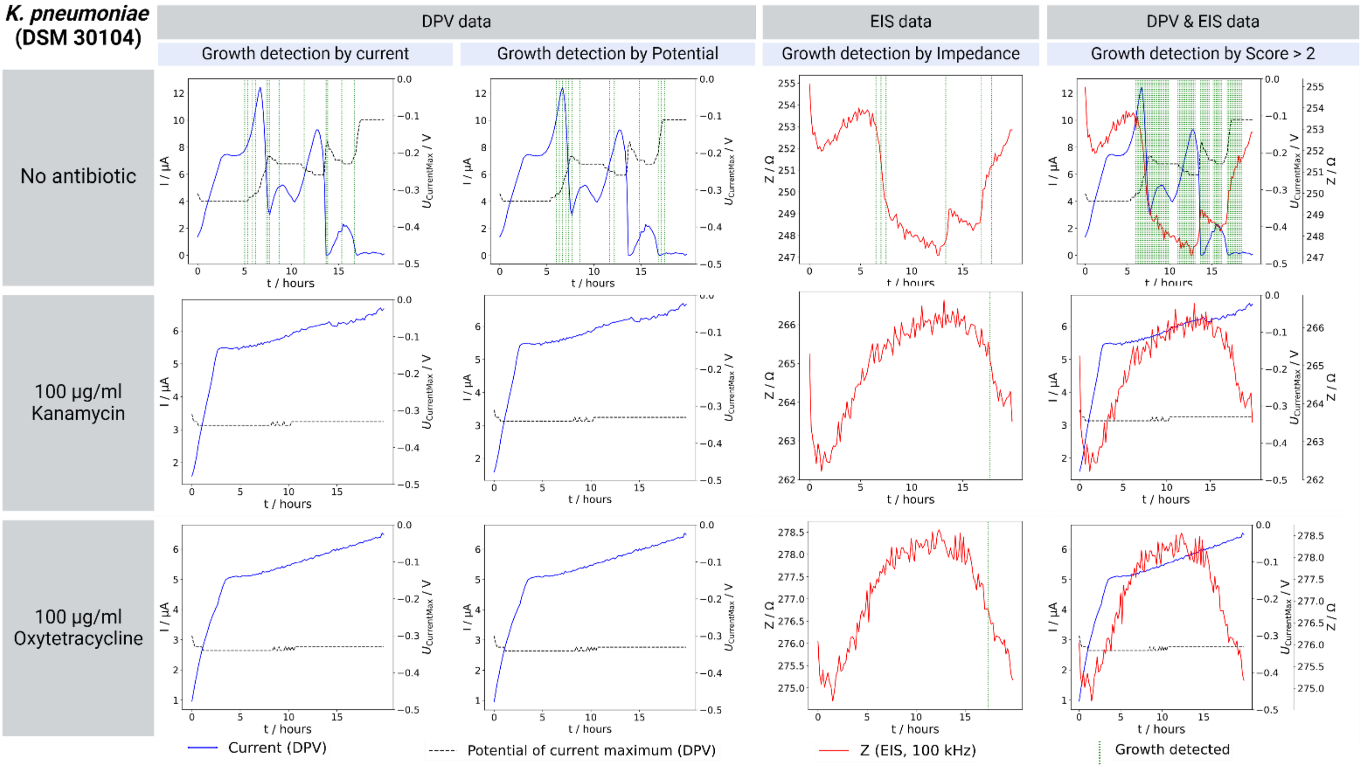

Figure S6. Electrochemical measurement and bioinformatics analysis for antibiotic susceptibility of *Klebsiella pneumoniae* (DSM 30104). Bacterial growth of *K. pneumoniae* with 100 µg/ml kanamycin, 100 µg/ml oxytetracycline, or without antibiotic was measured by differential pulsed voltammetry (DPV) and electrochemical impedance spectroscopy (EIS) in aerobic test device with an IS-C screen printed electrode. The plots show one of three representative replicates with evidence of growth by different parameters (current (*I*), potential (*U*), impedance (*Z*) and combined method (score)).

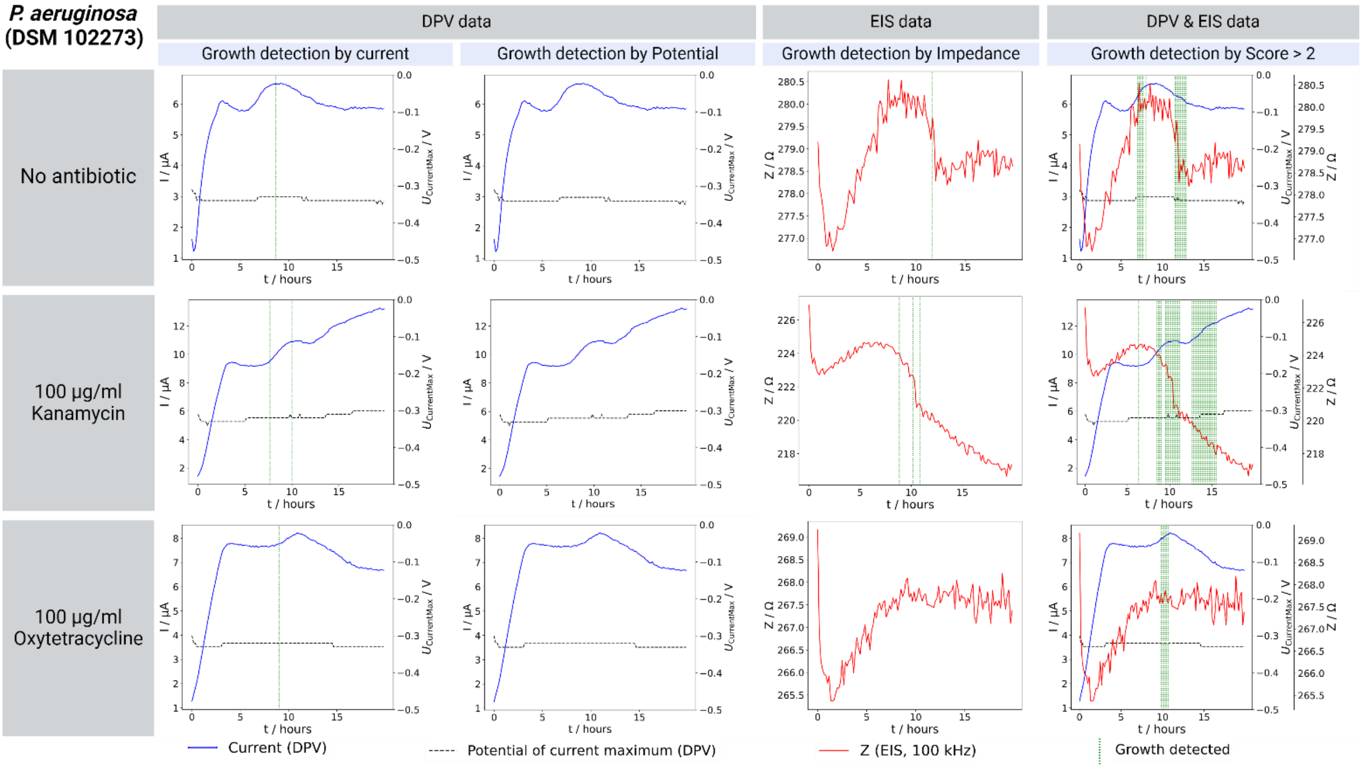

Figure S7. Electrochemical measurement and bioinformatics analysis for antibiotic susceptibility of *Pseudomonas aeruginosa* (DSM 102273). Bacterial growth of *P. aeruginosa* with 100 µg/ml kanamycin, 100 µg/ml oxytetracycline, or without antibiotic was measured by differential pulsed voltammetry (DPV) and electrochemical impedance spectroscopy (EIS) in aerobic test device with an IS-C screen printed electrode. The plots show one of three representative replicates with evidence of growth by different parameters (current (I), potential (U), impedance (Z) and combined method (score)).

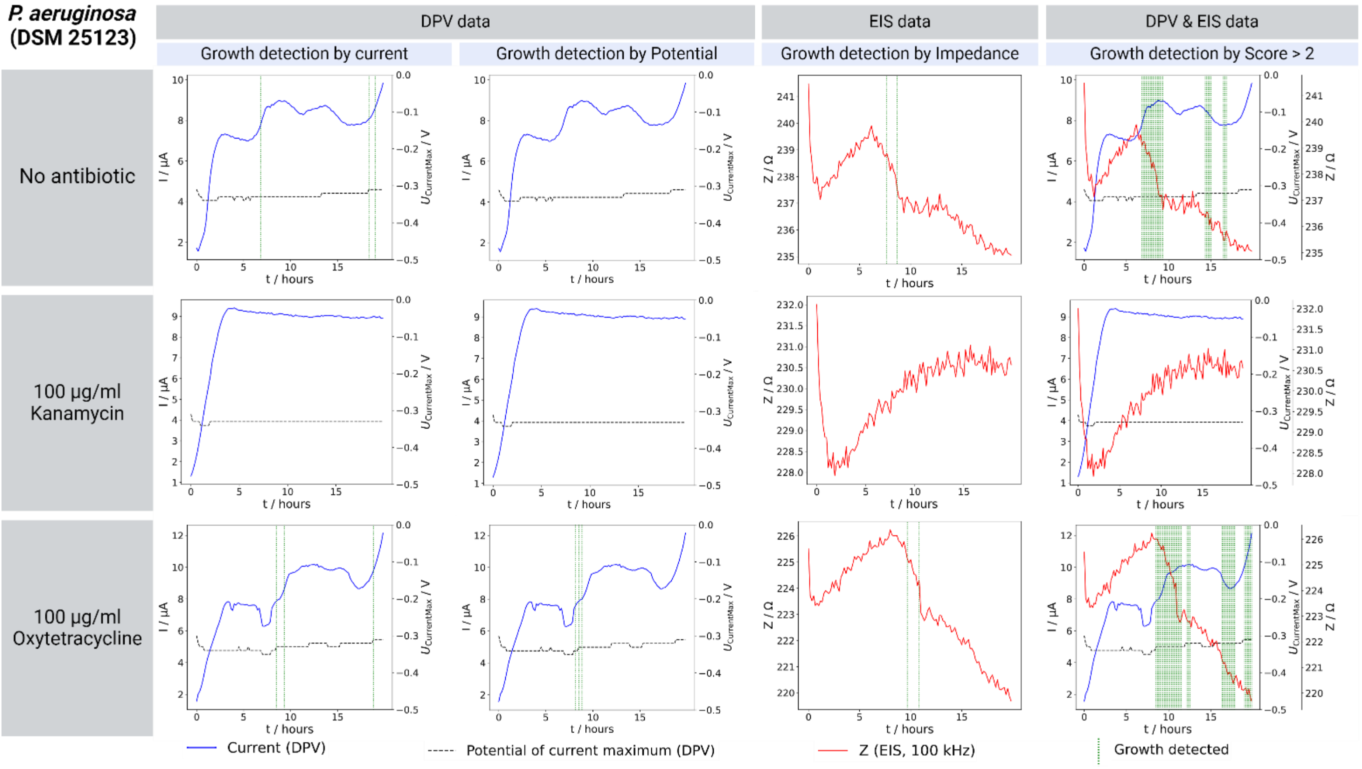

Figure S8. Electrochemical measurement and bioinformatics analysis for antibiotic susceptibility of *Pseudomonas aeruginosa* (DSM 25123). Bacterial growth of *P. aeruginosa* with 100 µg/ml kanamycin, 100 µg/ml oxytetracycline, or without antibiotic was measured by differential pulsed voltammetry (DPV) and electrochemical impedance spectroscopy (EIS) in aerobic test device with an IS-C screen printed electrode. The plots show one of three representative replicates with evidence of growth by different parameters (current (*I*), potential (*U*), impedance (*Z*) and combined method (score)).

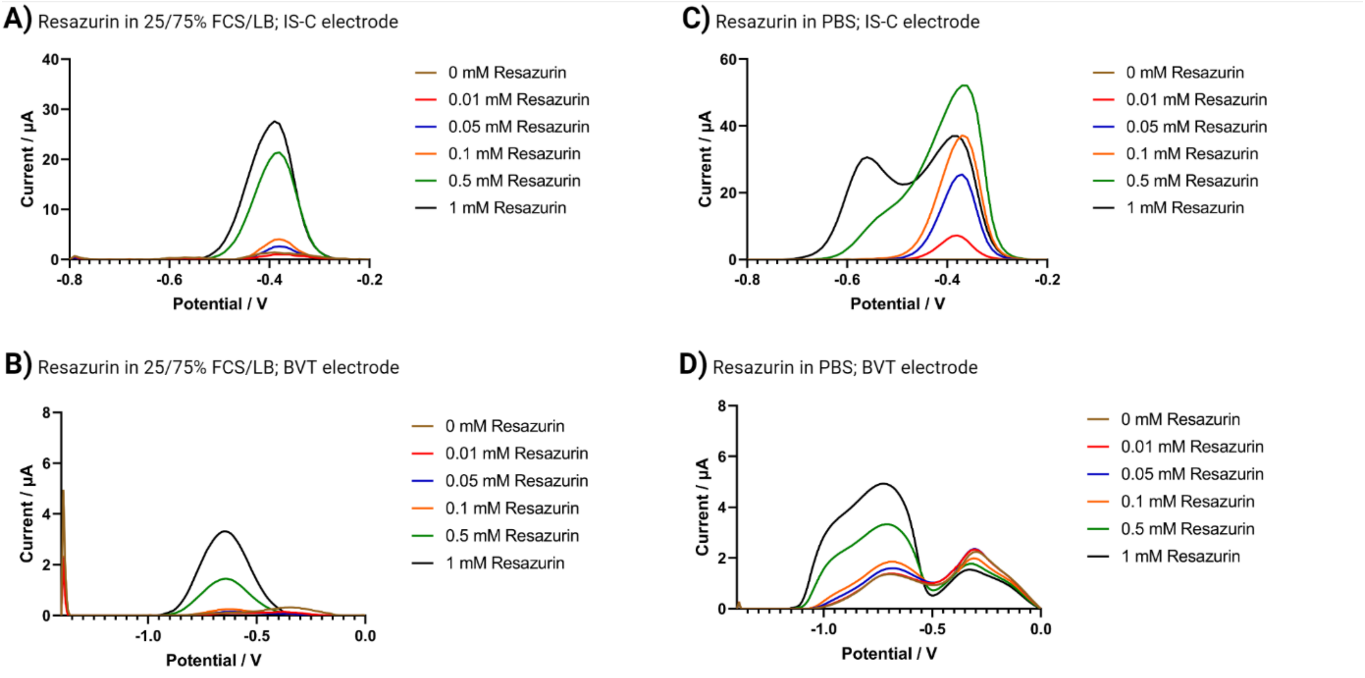

Figure S9. Differential pulse voltammetry (DPV) graphs of resazurin. DPV measurement of Resazurin on IS-C (graphite) and BVT (gold)

electrodes at different concentrations in 25/75% FCS/LB (A, B) or PBS (C, D). Values are shown as mean of (IS-C and BVT) 2 devices á 3 measurements.

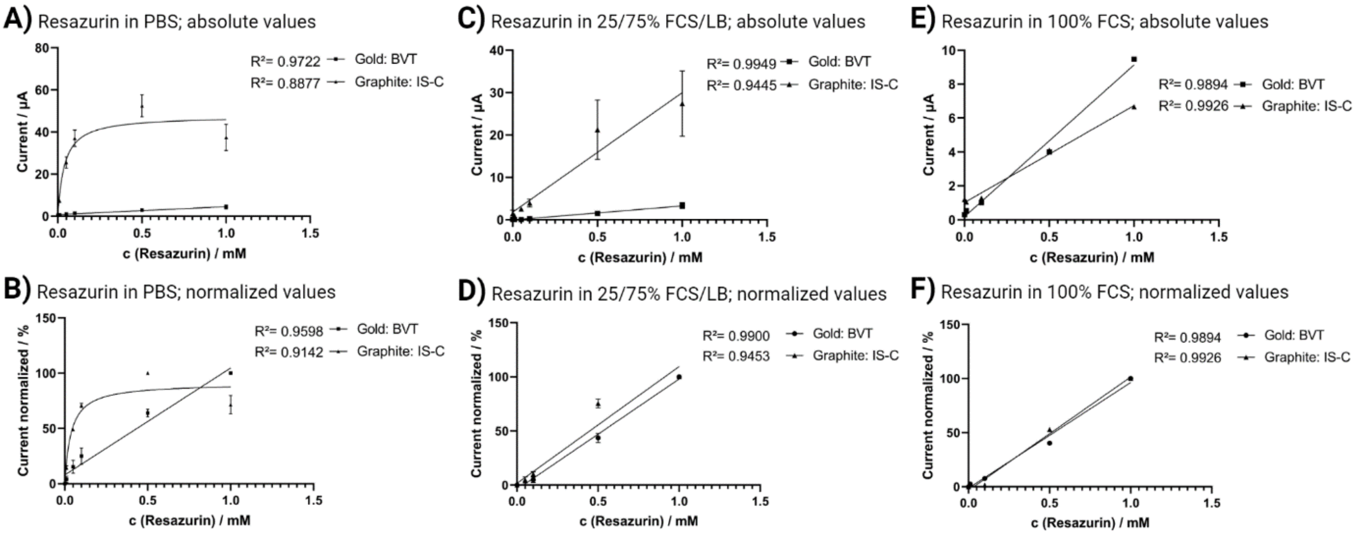

Figure S10. Concentration dependent signals of differential pulse voltammetry (DPV) for resazurin. Measurement of Signal on IS-C (graphite) and BVT (gold) electrodes at different concentrations in PBS (A and B), 25/75% FCS/LB (C and D) or 100% FCS (E and F). Current intensities of concentration dependent peak are shown as absolute values (A, C and E) or normalized for each electrode (B, D and F; 0 = lowest value, 100 = highest value). Values are shown as mean ± SD of (IS C and BVT) 2 devices á 3 measurements each (100% FCS only 1 device á 1 measurement). Linear regression or non-linear regression (IS-C in PBS) were performed with GraphPad Prism 8.

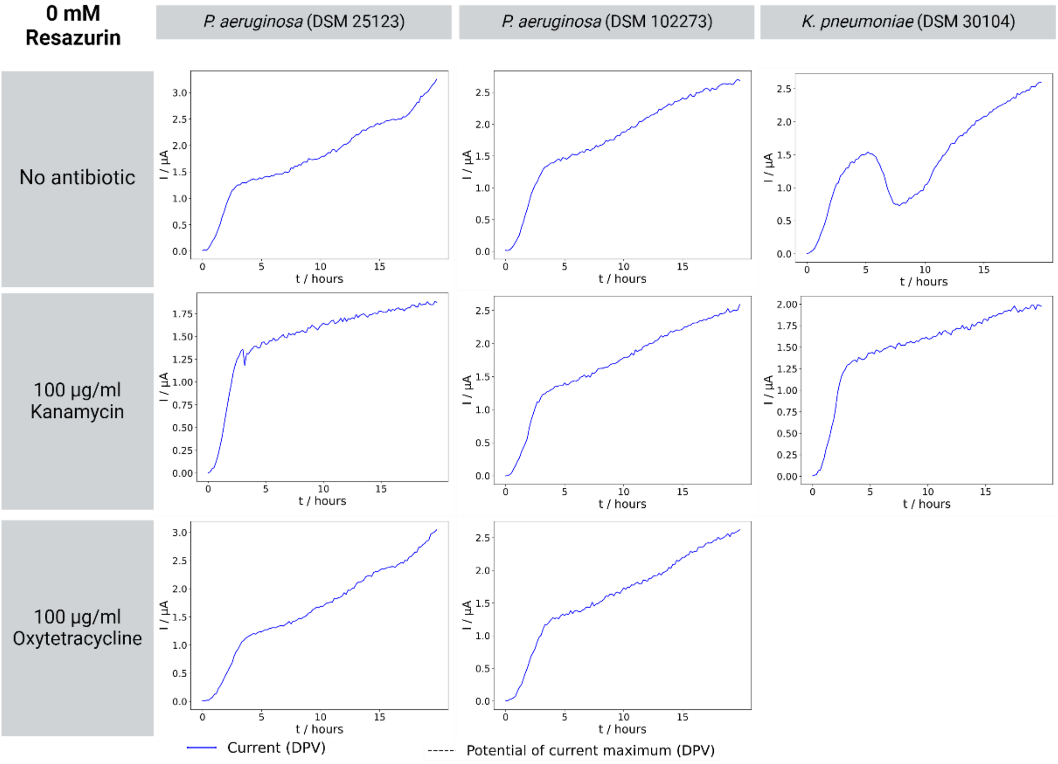

Figure S11. Differential pulsed voltametry (DPV) measurement without Resazurin. Current intensity (I) measured by DPV at – 0.3 V for *P. aeruginosa* (DSM 25123), *P. aeruginosa* (DSM 102273) and *K. pneumoniae* (DSM 30104) without the supplementation of Resazurin. Bacterial strains were treated with 100 µg/ml kanamycin, 100 µg/ml oxytetracycline, or without antibiotic in an aerobic test device with an IS-C screen printed electrode.

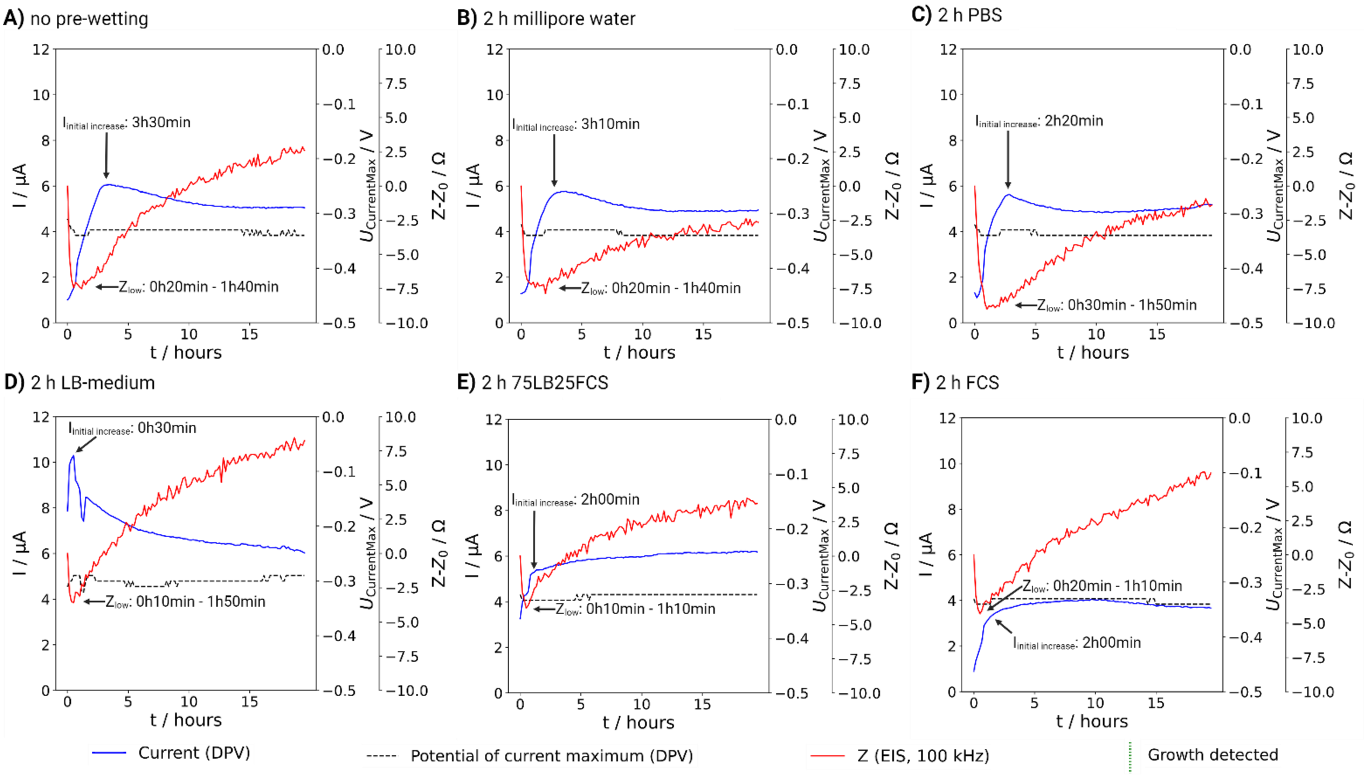

Figure S12. Influence of pre-treatment of the electrodes on the time to reach equilibrium. Aerobic one-chamber devices (n = 1) were pre-incubated

2 h with A) none, B) millipore water, C) PBS, D) LB-medium, E) 75% LB 25% FCS or F) 100% FCS prior to loading with 25/75% FCS/LB containing 0.2 mM Resazurin (t = 0 h). Time for initial increase of current ($I_{initial\ increase}$) and time for initial low of impedance (Z_{low}) indicated with arrows for each pre-treatment.

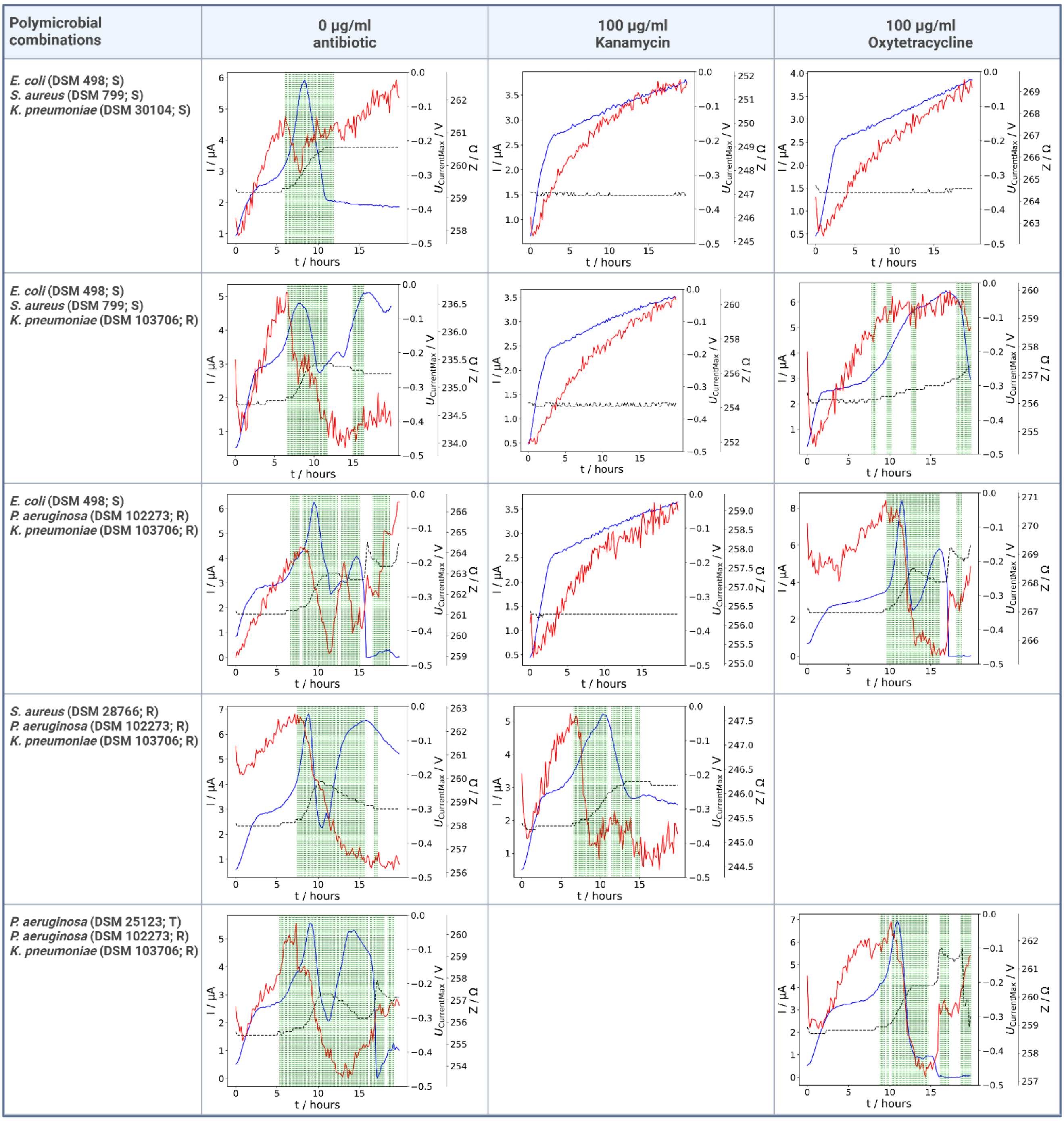

S: Sensitive R: Resistant T: High tolerance

Figure S13. Evaluation of artificial polymicrobial infections in human plasma by the combined method in the 3D printed prototype. Measurements (n = 1) were performed for combinations of three strains randomly selected from the available pathogens used in this study to match samples from 3 sensitive (S) to 3 resistant (R) strains for both antibiotics.

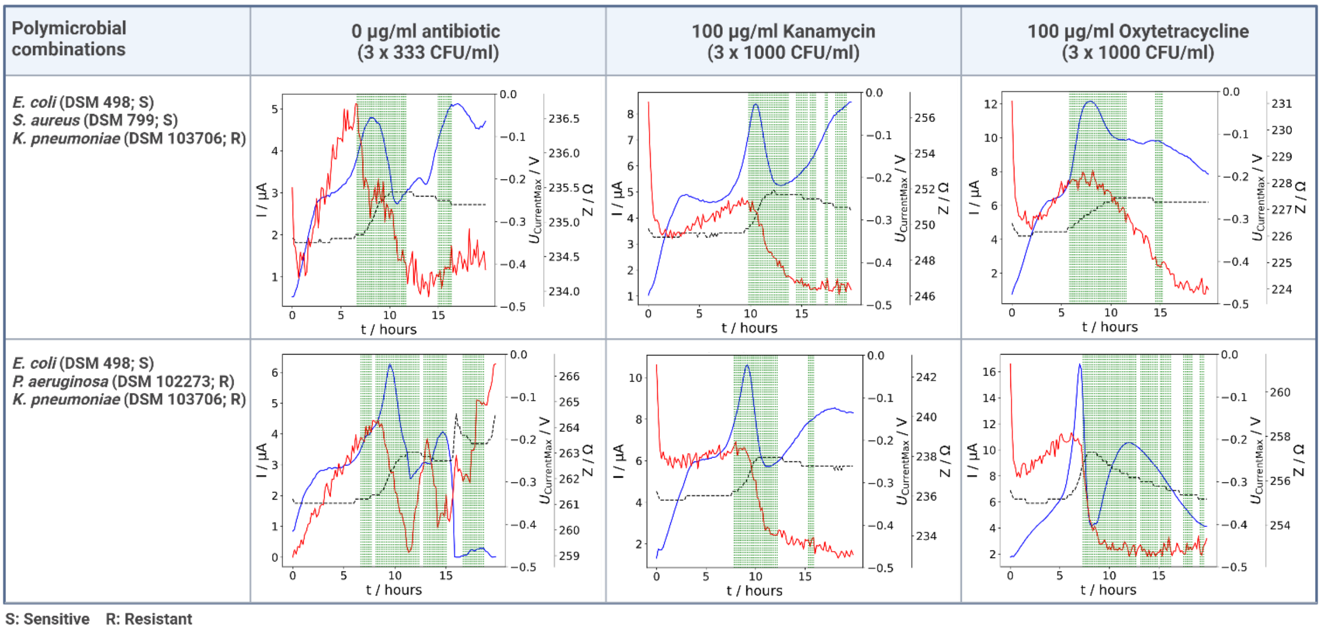

Figure S14. Evaluation of artificial polymicrobial infections in human plasma by the combined method in the 3D printed prototype. Measurements (*n* = 1) were performed for combinations of three strains according to Figure S13. Initial bacteria concentration of treated samples were tested with 1000 CFU/ml per strain to match non-polymicrobial AST experiments.

A) Broth microdilution

Concentration antibiotic	100 µg/ml	10 µg/ml	1 µg/ml	0.1 µg/ml	0.01 µg/ml
S.aureus (DSM 799) Kanamycin	✓	✓	✓	✗	✗
K. pneumoniae (DSM 30104) Kanamycin	✓	✓	✓	✓	✓
S.aureus (DSM 799) Oxytetracycline	✓	✓	✓	✗	✗
K. pneumoniae (DSM 30104) Oxytetracycline	✓	✓	✓	✓	✓

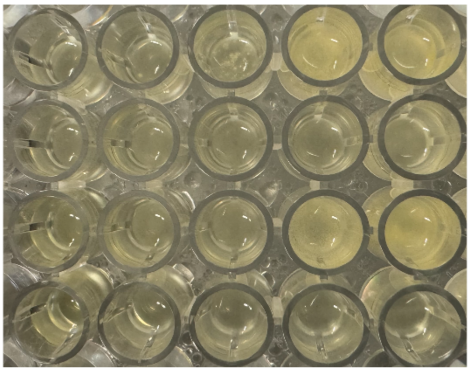

✓ Sensitive / no growth observed ✗ Resistant / growth observed

B) MIC by 3D printed prototype

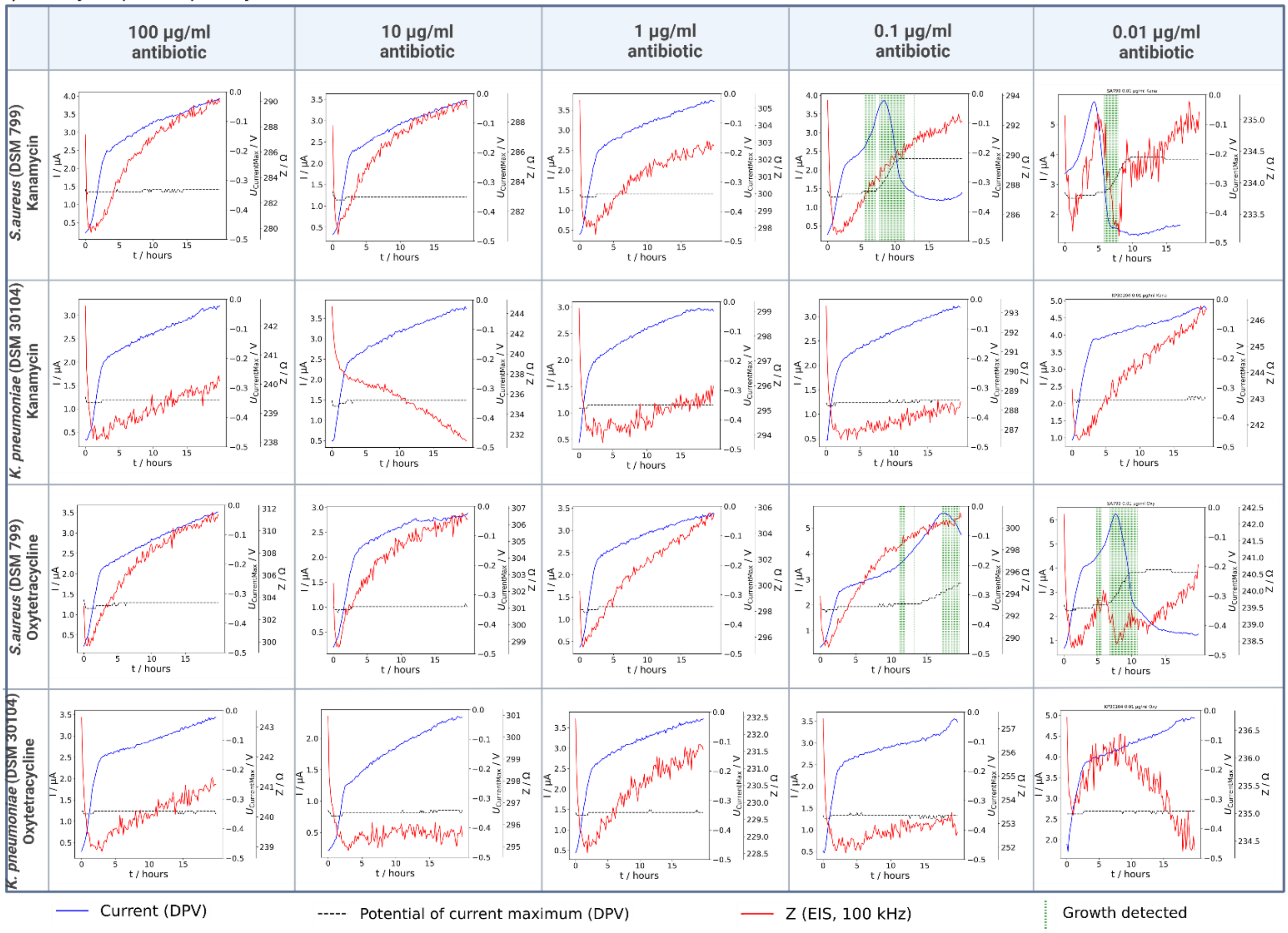

Figure S15. Minimum inhibitory concentration (MIC) measurement by A) broth microdilution and B) 3D printed prototype. Measurements (n = 1) were performed for 2 sensitive strains, *S.aureus* (DSM 799) and *K. pneumoniae* (DSM 30104), with different concentrations of kanamycin and oxytetracycline. Detection of growth for the electrochemical AST was performed with the combined method.

References

[1] B. Kowalska-Krochmal, R. Dudek-Wicher, *Pathogens* **2021**, *10*, 165.

[2] International Standard. ISO 20776-1, *Susceptibility Testing of Infectious Agents and Evaluation of Performance of Antimicrobial Susceptibility Test Devices. Part 1, Broth Micro-Dilution Reference Method for Testing the in Vitro Activity of Antimicrobial Agents against Rapidly Growing Aerobic Bacteria Involved in Infectious Diseases*, British Standards Institution, London, **2020**.

[3] H. A. Crosby, J. Kwiecinski, A. R. Horswill, in *Analytical and Bioanalytical Chemistry*, **2016**, pp. 1–41.

[4] A. E. Deller, B. M. Hryniewicz, C. Pesqueira, R. P. Horta, B. J. G. Da Silva, S. Weheabby, A. Al-Hamry, O. Kanoun, M. Vidotti, *Polymers* **2023**, *15*, 739.

[5] Ø. Ø. Dalheim, S. Steen, *Journal of Ocean Engineering and Science* **2020**, *5*, 333.

[6] I. Galani, E. Xirouchaki, K. Kanellakopoulou, G. Petrikkos, H. Giamarellou, *Clinical Microbiology and Infection* **2002**, *8*, 579.
